# Mechanical and mineralogical performance of sustainable cement composites with calcined palm oil leaf and calcined pine leaf Ash as supplementary cementitious materials

**DOI:** 10.1038/s41598-025-20196-2

**Published:** 2025-09-18

**Authors:** Mostafa Samadi, G. Murali, Leong Sing Wong, Norashidah Md Din, Isyaka Abdulkadir, Mohamed Abdellatief, Nor Hasanah Abdul Shukor Lim, Zelalem Mebrate

**Affiliations:** 1 Institute of Energy Infrastructure, Universiti Tenaga Nasional, Jalan IKRAM-UNITEN, Kajang, 43000 Selangor Malaysia; 2 Centre for Promotion of Research, Graphic Era (Deemed to be University), Clement town, Dehradun, India; 3 Department of Civil Engineering, College of Engineering, Universiti Tenaga Nasional, Jalan IKRAM- UNITEN, Kajang, 43000 Selangor Malaysia; 4 Department of Civil Engineering, Higher Future Institute of Engineering and Technology in Mansoura, Mansoura, Egypt; 5 Faculty of Civil Engineering, UTM Construction Research Center (CRC), University Teknologi Malaysia, Blok C09, 81310 Johor Bahru, Johor Darul Takzim Malaysia; 6 Department of Construction Technology and Management, Wollega University, Wollega, Ethiopia

**Keywords:** Palm oil leaf, Pine leaf, Supplementary cementitious materials, Strength, Microstructure, Sustainability, Chemistry, Engineering, Environmental sciences, Materials science

## Abstract

The construction sector exerts a profound environmental burden, largely attributable to cement production, which represents a major source of global carbon emissions. In pursuit of sustainable alternatives, this study explores the utilisation of agricultural by-products specifically calcined palm oil leaf ash (POLA) and calcined pine leaf ash (PLA) as novel supplementary cementitious materials (SCMs). Both ashes, when thermally processed, exhibit considerable potential to enhance the mechanical performance and sustainability of cementitious composites. An experimental programme was undertaken to evaluate mortars incorporating 5–30% POLA or PLA, in 5% increments, as partial cement replacements. Mechanical, microstructural, and phase-characterization studies were performed on both fresh and hardened states. Results demonstrated that high replacement levels of PLA (30%) severely compromised compressive strength, With reductions of 72.27%, 69.87%, and 67.64% at 7, 14, and 28 days, respectively. By contrast, equivalent POLA mixtures exhibited more moderate declines of 43.54%, 32.05%, and 32.72% over the same periods. At low incorporation (5%), both ashes fostered compact, homogeneous microstructures enriched in calcium silicate hydrate gel, reflecting robust pozzolanic activity and accelerated hydration. X-ray diffraction and Fourier-transform infrared spectroscopy confirmed these observations, revealing depletion of calcium hydroxide phases and significant modifications in Si-O-Si and O-H vibrational bands at optimal substitution levels. Conversely, excessive ash loadings impeded hydration, with persistent residues, peak broadening, and attenuated CH intensities. Overall, this study demonstrates the valorisation of calcined POLA and PLA as previously unreported SCMs, establishing their potential to reduce cement’s carbon footprint while maintaining microstructural integrity and mechanical viability at optimal dosages.

## Introduction

**Fig. 1:**
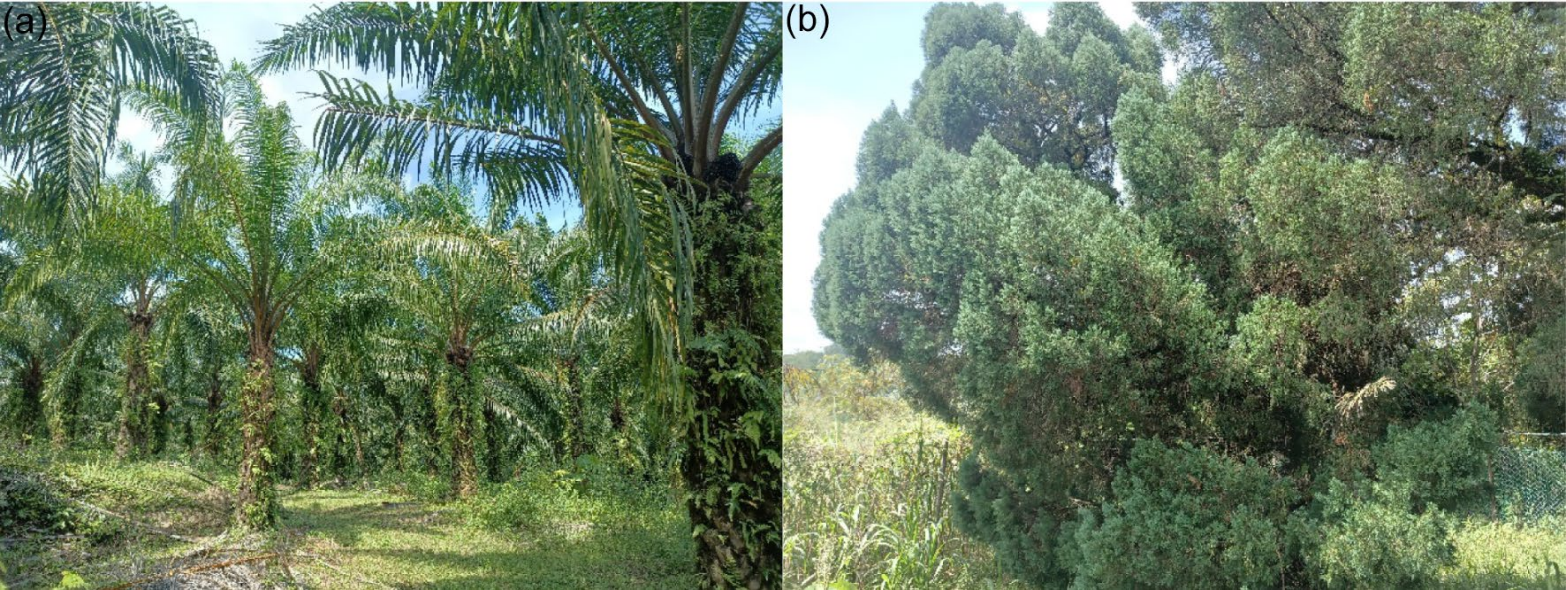
Tree (**a**) Palm oil and (**b**) Pine.

The global building sector, while serving as a core contributor to economic progress, is concurrently a major contributor to environmental concerns, largely attributed to the intensive use of Portland cement^1^. The production of cement accounts for approximately 8% of global CO₂ emissions^2^stemming from its high energy demands and the environmental drawbacks inherent in traditional production methodologies. In response to increasing global advocacy for sustainable construction practices, the exploration of environmentally sustainable alternatives to conventional cement has become a research priority^3^. One promising strategy involves incorporating supplementary cementitious materials (SCMs) extracted from the residual biomass and waste of industrial and agricultural origins^4,5^. Agricultural waste ashes, including palm oil leaf ash (POLA) and pine leaf ash (PLA), have demonstrated potential as sustainable SCMs due to their pozzolanic characteristics^6,7^. The incorporation of these materials contributes to lowering the carbon footprint of cement production while simultaneously promoting the sustainable management and valorization of biomass-derived waste and aligning with circular economy principles^8,9^. Malaysia, recognized as a leading contributor to the global palm oil industry, ranks as the second-largest exporter of crude palm oil worldwide, responsible for nearly 24% of total global output, trailing only behind Indonesia^10^. Similarly, pine leaf biomass from Malaysia’s forests can be converted into PLA through incineration. The palm oil tree and pine tree, from which palm oil leaves and pine leaves are obtained in Malaysia, are illustrated in the Fig. 1. Utilizing these agricultural wastes not only aids in waste management but also supports the partial replacement of cement, promoting environmentally sustainable construction practices. The use of POLA and PLA as SCMs offers both environmental and engineering advantages. These ashes demonstrate pozzolanic activity by chemically interacting with calcium hydroxide to generate calcium silicate hydrate (C-S-H) phases, which contribute to the improvement of the mechanical strength and durability of concrete. POLA, rich in silica and alumina, and PLA, containing silica, calcium oxide, and potassium, improve the strength and longevity of concrete^11^. By partially replacing ordinary Portland cement (OPC) with POLA and PLA, carbon emissions are reduced, waste is valorized, and concrete gains improved workability, chemical resistance, and durability, supporting sustainable, low-carbon construction practices^12^.

A growing body of research has demonstrated the potential of various agro-waste ashes as sustainable SCMs for enhancing concrete properties. Amin et al^13^. found that combining 2.5-5% NCSA With 20% palm leaf ash yielded superior 90-day compressive strengths (161.7–170.1 MPa), although higher replacement levels (e.g., 10% NCSA + 30% palm leaf ash) led to a significant slump flow reduction of 14.5%, indicating a trade-off between strength and workability. In comparison, Bakar et al^14^. demonstrated that the use of mussel shell ash With POLA can also contribute to strength enhancement, achieving 50 MPa With just 1% and 1.5% substitutions, respectively. These results, although from a different concrete class, underscore the efficacy of low-volume ash replacements in improving mechanical performance. Gharibi et al^15^. further expanded the range of viable ashes by investigating pine ash and conifer leaf ash, revealing that 15–20% replacement levels significantly lowered thermal conductivity (by up to 26%) and water absorption, thus making these ashes suitable for thermally efficient construction applications. Similarly, Khan et al^16^. observed that tobacco stem ash (TSA) at 7.5–15% facilitated early hydration, reduced porosity, and improved microstructure through increased portlandite and C-S-H formation. However, the need for precise control over ash content becomes apparent when comparing Patnaik et al^17^., who noted reduced workability and marginal compressive strength loss using Bermuda grass ash (BGA), largely due to insufficient pozzolanic activation from suboptimal calcination (600 °C). Frías et al^18^. validated the potential application of bamboo leaf ash (BLA) as an effective pozzolanic material in composite cement formulations (10–20% replacement), with strength comparable to control specimens and no dimensional instability, although higher water demand was noted. The conclusions of Bhutto et al^19^. and Kanning et al^20^. further support the efficacy of banana leaf ash (BALA) in enhancing both compressive strength (by 10–40%) and durability. Yet, Islam et al^21^. pointed out that excessive BALA content may reduce strength as a result of decreased C-S-H and portlandite development, highlighting the need to optimize dosage. In the realm of ultrahigh-strength concrete, Agwa et al^22^. established that sugarcane leaf ash calcined at 700 °C enhanced compressive strength (162.5 MPa at 20% replacement) but reduced slump flow with higher dosages, mirroring trends seen with other ashes.

Similarly, Kareem et al^23^. noted a strength decline at high dosages of cashew leaf ash (CLA), although a 5% substitution outperformed the control at 90 days, aligning with the trend that low-to-moderate replacements optimize performance while maintaining durability thresholds. The conclusions by Bunyamin et al^24^. reinforce the strength benefits of a 10% replacement level using *Cymbopogon nardus*leaf ash, beyond which workability deteriorates, reflecting the limitations of high ash content. Lastly, Charime et al^25^. demonstrated that 8–16% cane ash (CA) not only improved workability (19.5–23.0 cm) and modulus of elasticity (23.6–24.9 GPa) but also promoted microstructural densification and reduced porosity over time due to enhanced secondary C-S-H and reduced ettringite formation. Agwa et al. examined ultrahigh-strength concrete with sugarcane leaf ash (SLA) replacing cement at 10–30% by weight and found that 20% SLA calcined at 700 °C yielded the highest compressive strength of 162.5 MPa. A progressive diminished slump flow spread was observed With increasing levels of SLA used as a partial substitution of cement. Specifically, the recorded slump flow values were 454 mm, 438 mm, and 422 mm for mixtures comprising 10%, 20%, and 30% SLA, respectively, whereas the control mixture (0% SLA) exhibited a diameter of 436 mm. Murali et al^26^. examined the use of *Dillenia suffruticosa* (DS) and *Acacia auriculiformis* (AA) leaf ashes as supplementary cementitious materials. Replacing cement with DS ash (5%−40%) increased both initial (83–98 min) and final setting times (124–155 min). AA ash showed similar effects, With setting times ranging from 88 to 108 min for initial and 131 to 170 min for final. Both ashes, containing calcium, K₂O, and Na₂O, enhanced portlandite and C-S-H formation, improving mortar strength. However, at 40% ash content, mortar density decreased and porosity increased due to poor packing of irregular ash particles.

Agricultural ashes demonstrate pozzolanic activity, yet their performance is predominantly driven by core influences such as calcination temperature, degree of particle size reduction, and the extent of cement partial substitution. Typically, replacement levels between 5% and 20% are optimal for improving the mechanical and durability characteristics of composite cementitious system without compromising workability. When appropriately selected and processed, these ashes show considerable potential as sustainable, high-performance materials in construction, consistent with worldwide strategies for lowering greenhouse gas emissions. Accordingly, this research investigates the integration of calcined POLA and calcined PLA into cementitious composites, assessing their effectiveness in improving the properties of construction materials and advancing sustainability in the construction industry. High-temperature calcination improves the reactivity of POLA and PLA by removing residual organics, increasing amorphous silica phases, and enhancing surface texture, which together accelerate beneficial reactions with cement hydration products and strengthen the composite’s internal structure. In this study, pine and palm oil leaves, abundantly available in Malaysia, were collected, sun-dried to remove surface moisture, and then subjected to controlled open-air combustion in a well-ventilated environment (ambient temperature 26 °C) with adequate airflow to ensure complete burning. Inadequate airflow during biomass combustion, even under ventilated conditions, can cause incomplete oxidation, resulting in residual carbon, reduced amorphous silica content, and diminished pozzolanic reactivity. This not only produces ash of inconsistent quality but also increases the risk of undesirable emissions. Ensuring sufficient airflow is therefore critical to achieve complete combustion, stable ash chemistry, and high reactivity for cementitious applications. The burning process continued until the biomass was fully converted into ash, which was subsequently collected and stored for calcination. Globally, large volumes of biomass residues (e.g., rice husk, sugarcane bagasse, palm oil fronds, pine needles) are similarly processed through combustion and calcination, with their incorporation into cementitious systems shown to lower cement demand, mitigate CO_2_ emissions, and promote circular economy practices by transforming waste into value-added resources.

## Research significance

This study constitutes a pioneering investigation into the application of calcined POLA and calcined PLA as blended SCMs within cementitious systems. Departing from earlier research that considered these agro-waste ashes in isolation, this work provides a novel assessment of their integrated effects on the mechanical, rheological, and microscopic architecture of cementitious matrices. A structured experimental methodology comprising compressive strength evaluations, scanning electron microscopy (SEM), and X-ray diffraction (XRD) analyses was adopted to investigate optimal substitution levels that enhance performance while contributing to the reduction of cement consumption. The significance of this study lies in its innovative ash strategy and its in-depth analysis of material interactions across varying replacement levels, thereby filling a lack of comprehensive insight in existing research. Moreover, by promoting the utilization of agricultural residues typically regarded as waste, the research advances sustainable construction practices and supports key environmental goals, including lowering carbon emissions, conserving raw materials, and fostering circular economy principles in the context of the Malaysian construction sector. This work introduces a new approach by combining calcined POLA and calcined PLA as dual-purpose cement additives, focusing on their interactive behavior within cementitious systems to improve cement composite performance and promote eco-friendly alternatives in construction.

## Experimental design

### Materials

**Fig. 2:**
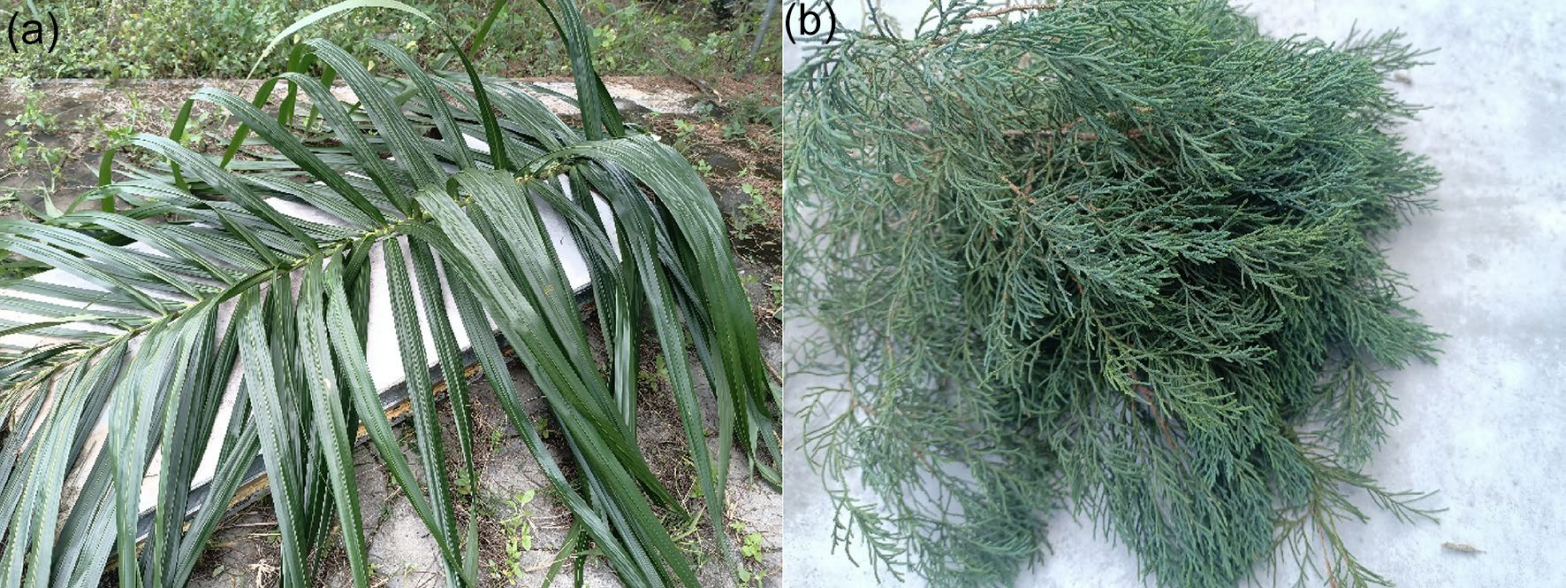
Leaves (**a**) Palm oil and (**b**) Pine.

- The Ordinary Portland Cement (OPC) employed in this investigation was used, exhibiting a bulk density of 1386 kg/m^3^ as determined through experimental measurement.
- The fine aggregate employed in this research was river sand obtained locally, exhibiting a maximum particle dimension of 2.34 mm, fineness modulus of 2.4 and a specific gravity value of 2.64. Sand in concrete should be free from dust, clay, silt, and impurities that can weaken the bond between cement and aggregate, ultimately reducing cement composite strength.
- In this study, the palm oil and pine leaves were collected in Malaysia (Fig. 2). Calcined ashes originating from palm oil and pine leaves were utilized as partial replacements for cement in the composite formulation.
- The workability of the cement composite was enhanced through the incorporation of a superplasticizer into the mix. A dose of 0.4% by the weight of cement was employed for all the mixes. Dynamon NRG 1010 is a high-performance, high-range water-reducing and hardening-accelerating superplasticiser engineered by Mapei. Possessing a density of 1.05 ± 0.02 g/cm³ and presented as a pearlescent liquid, it markedly enhances workability while effecting substantial reductions in water demand. Its principal merit lies in the pronounced acceleration of early-age strength development, even under low-temperature conditions, thereby facilitating rapid demoulding and optimised production efficiency.

#### Ash synthesis and processing methodology

**Fig. 3:**
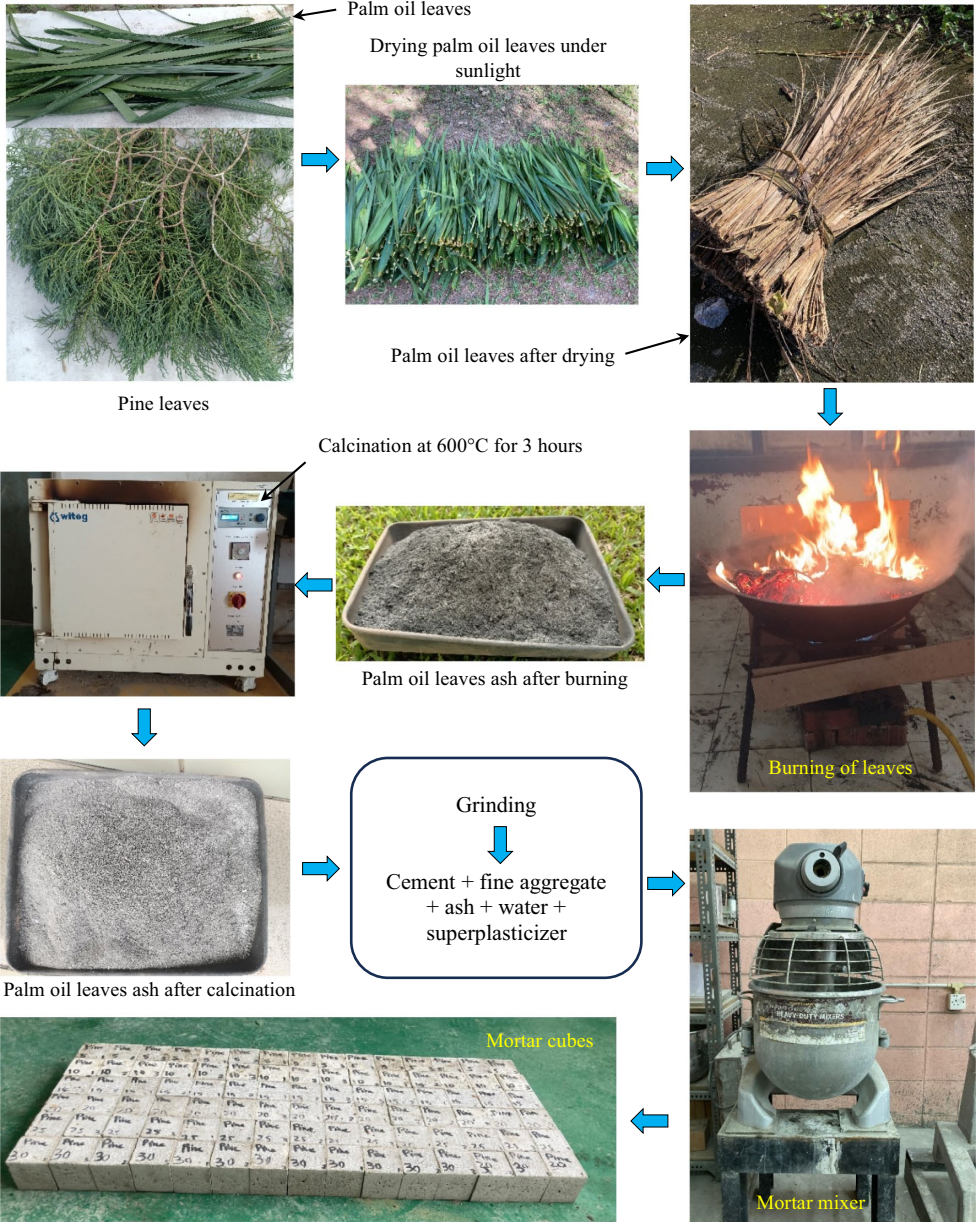
Ash production and preparation of cement composite specimens.

Palm oil and pine leaves obtained from a common locality in Malaysia were processed to derive ash, following the procedural sequence outlined in Fig. 3. The collected pine leaves and palm oil leaves were first dried under direct sunlight to remove surface moisture. Subsequently, the dried leaves were subjected to open-air combustion under controlled conditions in a well-ventilated room With an ambient temperature of approximately 26 °C, ensuring adequate airflow during burning. The combustion was continued until the leaves were fully reduced to ash, which was then collected and stored for subsequent calcination. The thermal decomposition of cellulose, hemicellulose, and lignin is essentially complete below 550 °C, resulting in the formation of a stable ash matrix at 600 °C^13,15,27,28^. At this critical temperature, the proportion of amorphous silica is maximised, thereby enhancing the pozzolanic reactivity of the ash. Calcination at higher temperatures, particularly beyond 600 °C, induces the crystallisation of silica into less reactive polymorphs such as quartz and cristobalite, which markedly diminishes its efficacy in cementitious systems. Furthermore, maintaining the calcination process at 600 °C not only ensures the complete elimination of residual organics but also achieves this With reduced energy input, consistent With principles of sustainable processing. Accordingly, 600 °C represents the optimal calcination temperature, whereas 700 °C constitutes the threshold beyond which reactivity declines due to phase crystallisation^29,30^. Accordingly, 600 °C was identified as the optimum calcination temperature, providing a balance between compositional purity and the retention of high pozzolanic activity in POLA and PLA. This calcination protocol is consistent with established practices reported in prior studies for producing reactive ash from lignocellulosic residues^23,27,31^. Following the calcination process, the resulting ash was subjected to mechanical pulverization using a ball mill, followed by sieving to achieve a consistent particle size distribution below 75 μm. The refined ash was subsequently incorporated as a partial cement substitute and systematically combined with fine aggregate based on predefined mix ratios for the preparation of standardized mortar cubes.

#### Visual appearance of Ash prior to and following calcination

**Fig. 4:**
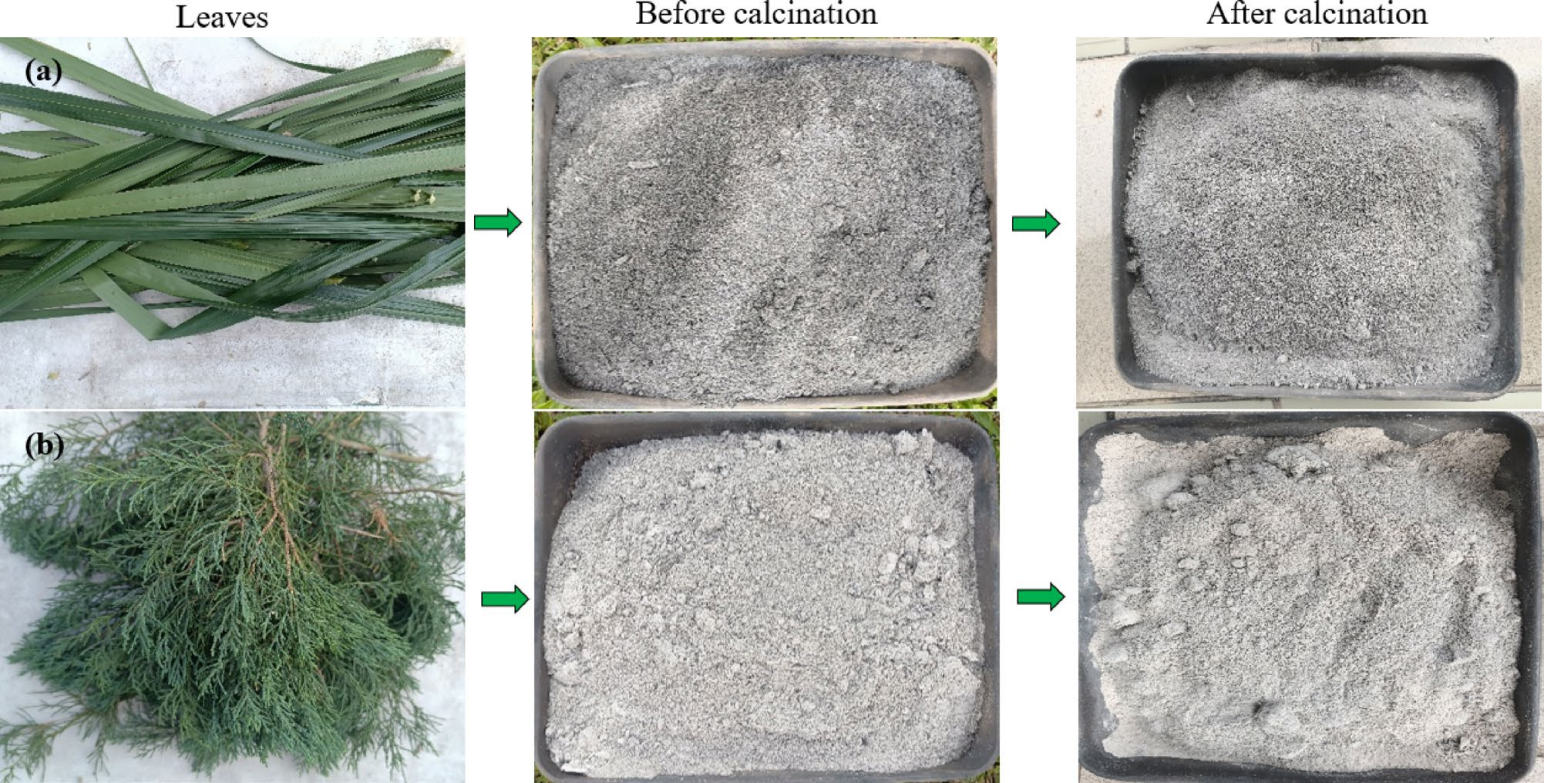
Comparative observation of leaf ash coloration prior to and following calcination (**a**) Palm oil and (**b**) Pine.

Figure 4 presents the morphological transformations and chromatic variations observed in palm oil leaf and pine leaf residues because of a controlled calcination treatment, highlighting the differences between their pre- and post-calcination conditions. Prior to calcination, POLA exhibits a dark gray to black color (Fig. 4a), indicating incomplete combustion and the presence of carbonaceous residues. After calcination, the ash turns lighter gray, reflecting improved combustion, reduced residual carbon, and a likely increase in purity and amorphous silica content favorable for pozzolanic activity in cementitious applications. The uncalcined PLA shows a grayish-black color (Fig. 4b), slightly lighter than uncalcined POLA, likely due to differences in lignin and cellulose content. After calcination, it turns nearly whitish-gray, indicating more efficient combustion and reduced organic residues. This lighter hue suggests higher reactive silica content and minimal unburnt carbon, reflecting improved ash quality. Other studies reported that the final product of the ash color derived from various agro-based materials, Khaki for peanut shell^32^Ecru for banana leaf^33^grey for tobacco stem^16^ and dark brown for Wallnut shell ash^34^light grey ash for *Dillenia suffruticosa*^26^ and light brown for *Acacia auriculiformis*^26^.

#### Particle size distribution of calcined leaf Ash

**Fig. 5:**
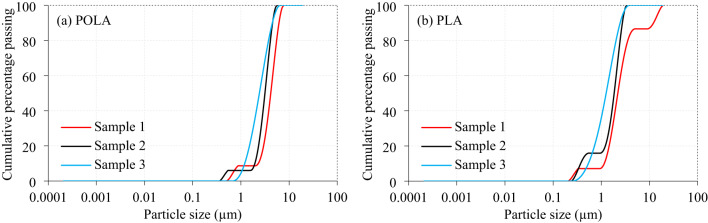
Particle size distribution of calcined ash (**a**) Palm oil leaf and (**b**) Pine leaf.

The calcined POLA and calcined PLA both exhibit particle sizes predominantly Within the range of 0.5–5 μm; however, calcined POLA demonstrates a markedly finer and more uniform distribution (Fig. 5), particularly evident in Sample 3. By contrast, calcined PLA, especially Sample 1, displays a broader and coarser size profile, indicative of greater heterogeneity in fineness. The narrower grading of calcined POLA not only signifies enhanced uniformity but also confers a higher specific surface area. This refinement increases the number of available reactive sites, thereby accelerating early pozzolanic reactivity through the rapid formation of nucleation centres for C-S-H phase growth. Consequently, calcined POLA fosters a denser and mechanically robust microstructure, characterised by reduced porosity and an improved pore structure, which collectively enhance the durability and long-term performance of the matrix. In contrast, the broader particle size distribution of calcined PLA may limit its early hydration kinetics due to the relatively lower surface area, yet it can still contribute beneficially to later-age strength through gradual pozzolanic activity. Overall, the finer grading and uniform distribution of calcined POLA provide it with a distinct advantage as a SCM, positioning it as a more reactive and efficient alternative to calcined PLA.

#### SEM analysis of calcined leaf Ash

**Fig. 6:**
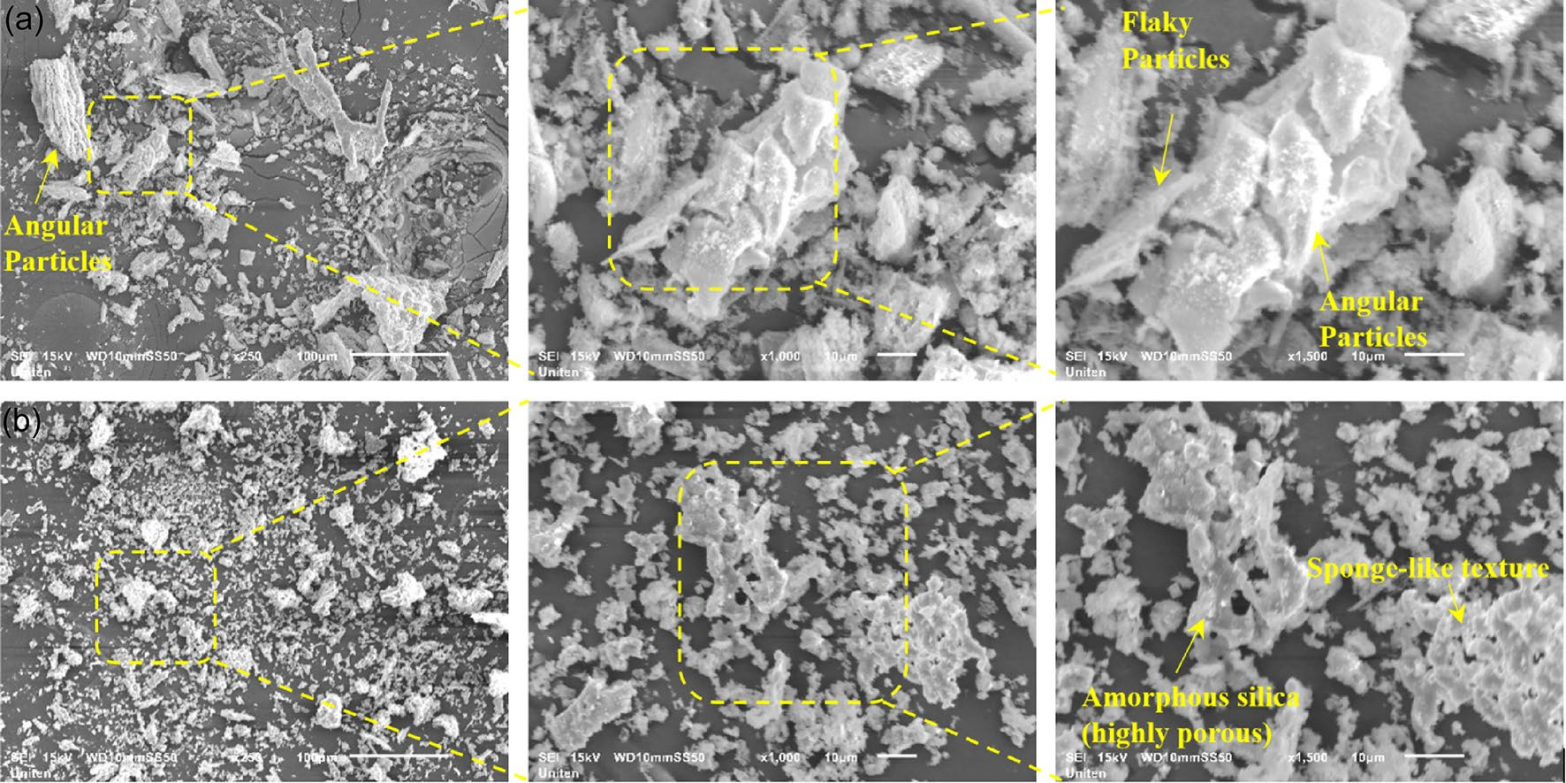
SEM analysis of calcined leaf ash (**a**) palm oil, (**b**) pine.

The surface morphologies of calcined POLA and calcined PLA were analysed using SEM at ×250, ×1000, and ×1500 magnifications to compare their microstructural features, as shown in Fig. 6. SEM analysis of the calcined POLA sample (Fig. 6a) reveals a heterogeneous morphology with porous, angular, and flaky particles. At ×250 magnification, coarse particles with fibrous carbonaceous remnants are observed, while higher magnifications (×1000 and ×1500) show stratified structures, indicating incomplete combustion. The rough texture suggests a high surface area favorable for pozzolanic activity, though unburnt residues may affect uniformity and filler efficiency without additional processing. SEM analysis of the calcined PLA sample reveals a finer (Fig. 6b), more uniform morphology with minimal fibrous residues, indicating more complete combustion and likely higher amorphous silica content. At higher magnifications, a sponge-like texture with micropores and micro-cracks is evident, suggesting enhanced water absorption and reactivity. These features support the material’s pozzolanic potential and effective integration into cementitious systems. Compared to calcined POLA, calcined PLA appears more suitable as a SCM, provided its chemical composition meets pozzolanic requirements. Other studies reported the particle size distribution of the other plant leaf ashes similar to our current study. For instance, Murali et al^26^. reported that *Dillenia suffruticosa* ash exhibits a coarse, crystalline structure with needle-like features, while *Acacia auriculiformis* ash shows a mixed surface texture varying from crystalline to glassy.

#### XRD analysis on calcined POLA and PLA

**Fig. 7:**
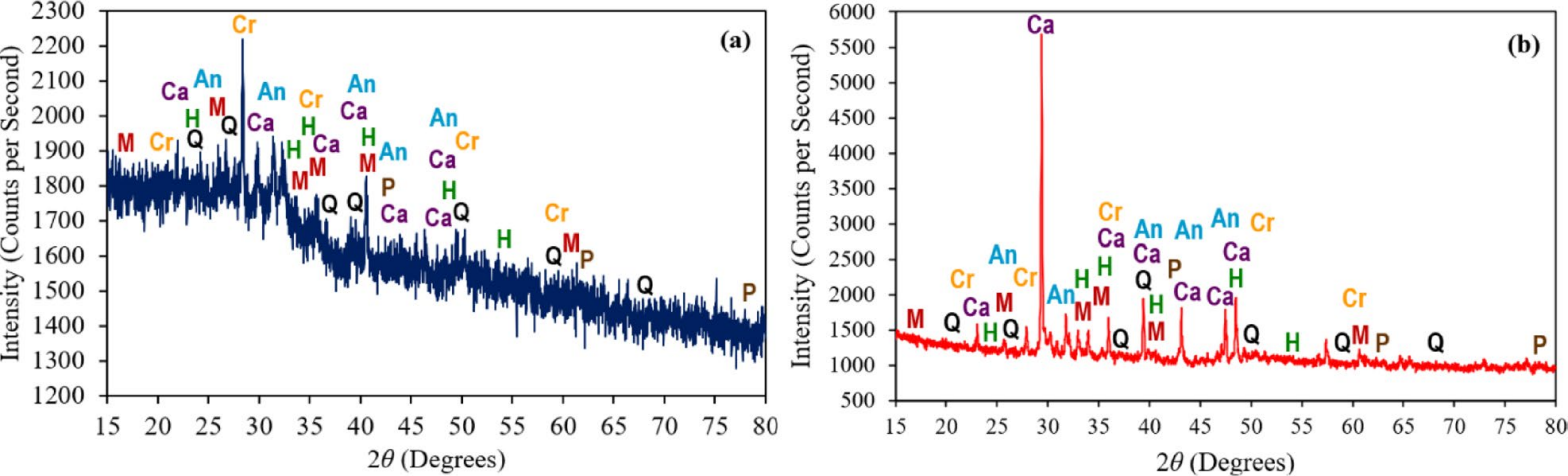
XRD test results for (**a**) calcined POLA and (**b**) calcined PLA. Note: Q = Quartz (SiO_2_), M = Mullite (Al_6_Si_2_O_13_), Ca = Calcite (CaCO_3_), P = Periclase (MgO), Cr = Cristobalite (SiO_2_ – high-temperature), An = Anhydrite (CaSO_4_), H = Hematite (Fe_2_O_3_).

Figures 7 (a) and (b) display the XRD of calcined POLA and calcined PLA, respectively. Notably, at 2θ angles of 23.0°, 29.4°, 36.0°, 39.5°, 43.2°, 47.5°, and 48.5°, prominent peaks corresponding to calcite (Ca) are evident in the calcined pine leaf ash, indicating a higher calcite content compared to the calcined POLA. This difference can be attributed to the greater calcium uptake by pine leaves from the soil relative to palm oil leaves. Upon calcination, the calcium compounds decompose to form calcium oxide, which subsequently reacts with atmospheric carbon dioxide during slow ambient cooling to form calcite. In contrast, the calcined PLA exhibits more pronounced peaks of mullite (M), hematite (H), and cristobalite (Cr), along With a higher degree of amorphousness in these mineral phases, as revealed by the XRD patterns. These findings suggest that, after undergoing calcination at 600 °C for 3 h, calcined POLA contains a greater proportion of amorphous pozzolanic minerals than calcined PLA. This observation aligns With studies indicating that calcination carried out at temperatures spanning 600 °C to 800 °C enhance the formation of amorphous phases in ash materials, thereby increasing their pozzolanic activity^35^. The observed mineralogical distinctions between calcined POLA and calcined PLA align with previously reported trends in the characterization of various biomass-derived ashes. For instance, the study^36^reported that the calcinated banana leaf ash with presence of cristobalite and calcium sulfate in aligns with Frías et al^18^., who identified similar crystalline phases at 2θ angles of 21.9°, 28.3°, and 36.1°, confirming its pozzolanic reactivity. Similarly, the XRD analysis of *Acacia auriculiformis* and *Dillenia suffruticosa* reveals diverse crystalline phases, including Montmorillonite, Hematite, Quartz, Biotite, and Calcite^26^. Notably, *Dillenia suffruticosa* ash shows a strong Biotite peak at 28.3°, while *Acacia auriculiformis* ash exhibits prominent Montmorillonite and Quartz peaks near 29.36°. The presence of Montmorillonite in *Acacia auriculiformis* ash enhances pozzolanic behavior by promoting lime interaction and densifying the cement matrix.

#### Materials mixing and specimen casting procedure

Thirteen cement composite formulations were developed, each incorporating varying dosages of ash for partial substitution of cement, as presented in Table 1. For each composition, triplicate specimens were prepared per testing day to ensure repeatability, and the mean values derived from these samples were used for data interpretation. The preparation procedure involved a two-stage mixing protocol: initially, the dry components cement, fine aggregates, and ash were blended in a mortar mixer for three minutes. This was followed by the gradual incorporation of a pre-blended water and superplasticizer solution, with mixing continued for an additional two minutes to achieve a uniform consistency. The fresh cement composite was then cast into standardized cube molds for compressive strength testing. Simultaneously, the same mixtures were evaluated for flowability using the flow table method, and their setting times (initial and final) were recorded. The reference mix, devoid of any ash substitution, was denoted as “CM.” Cement composites containing calcined POLA were labelled progressively as “POL-5,” “POL-10,” etc., based on the percentage of cement replaced. Likewise, mixes incorporating calcined PLA were categorized from “PL-5” to “PL-30” in accordance with the respective substitution levels.

**Table 1:** Mix proportion.

Mixture Id	Cement (kg/m^3^)	Fine aggregate (kg/m^3^)	Ash (%)	Type of ash	Ash (kg/m^3^)	W/C	SP (%)
CM	475.00	1072	0	-	0	0.4	0.4
POL-5	451.25		5	Calcined POLA	21.56		
POL-10	427.50		10		43.11		
POL-15	403.75		15		64.67		
POL-20	380.00		20		86.22		
POL-25	356.25		25		107.78		
POL-30	332.5		30		129.33		
PL-5	451.25		5	Calcined PLA	21.74		
PL-10	427.50		10		43.49		
PL-15	403.75		15		65.24		
PL-20	380.00		20		86.99		
PL-25	356.25		25		108.74		
PL-30	332.50		30		130.49		

#### Experimental testing

**Fig. 8:**
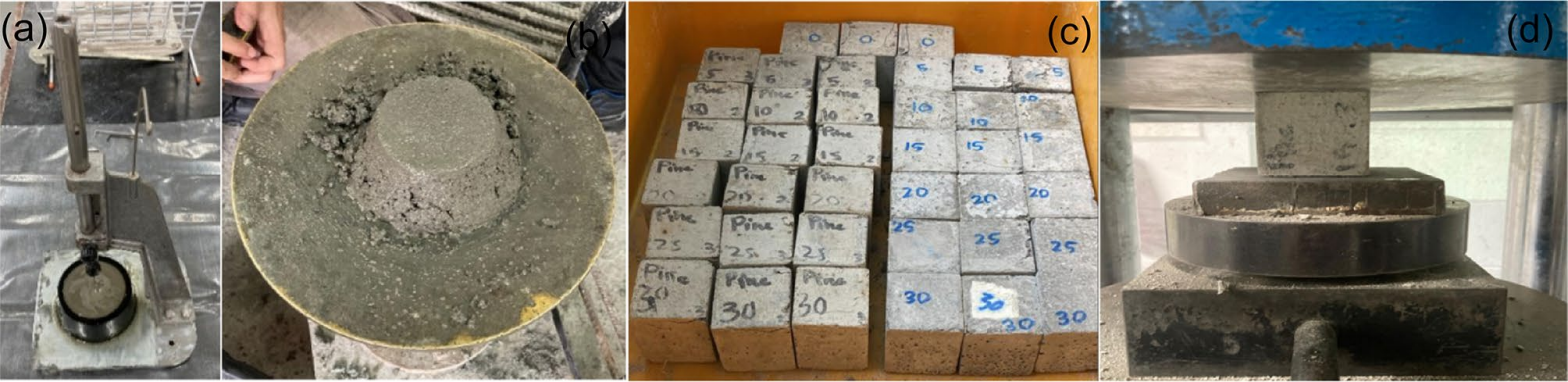
Testing methods and samples.

The density of both POLA and PLA was determined using a Quantachrome automatic pycnometer under standardized testing conditions. The setting times were measured in accordance with the standardized procedure detailed in ASTM C191^37^ (Fig. 8a). The flow behavior of the cement cement composite was assessed in accordance with the guidelines set forth in ASTM C1437-07^38^ (Fig. 8b). Compressive strength was evaluated using cube specimens With dimensions of 50 mm, which were prepared and tested following the procedures outlined in ASTM C109/C109M-21^39^ (Fig. 8c, d). For microstructural analysis, samples extracted from the tested specimens were examined utilizing Scanning Electron Microscopy (SEM), X-ray Diffraction (XRD), Thermogravimetric Analysis (TGA), and Fourier Transform Infrared Spectroscopy (FTIR).

## Discussion of results

The measured densities of calcined POLA and PLA were 2925 kg/m^3^ and 2591 kg/m^3^, respectively. The measured specific surface areas of OPC, POLA, and PLA were determined to be 0.315, 98.310, and 20.650 m²/g, respectively. The subsequent section presents a detailed discussion of the fresh and hardened properties of the cement composite.

### Setting time of cement composite

**Fig. 9:**
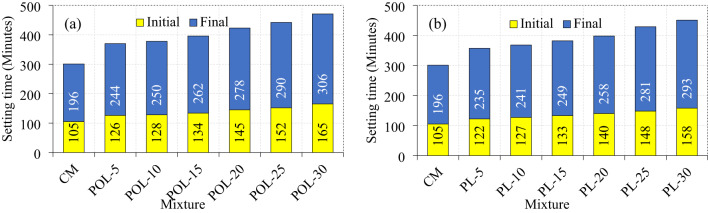
Setting time of fresh cement composite (**a**) calcined POLA and (**b**) calcined PLA.

The Figs. 9 illustrate the setting time behavior of cement composites incorporating varying proportions of calcined POLA and calcined PLA as partial cement replacements. The control mixture (CM) demonstrated the shortest setting intervals, With initial and final setting times recorded at 105 and 196 min, respectively. The study^26^ reported that the initial and final setting times of the cement composite were 80 and 122 min, respectively. Progressive substitution of cement With calcined POLA led to a steady increase in setting durations, With the initial setting time extending from 126 min at 5% replacement to 165 min at 30%, and the final setting time ranging from 244 to 306 min over the same substitution levels. A similar incremental pattern was observed in calcined PLA-based mixtures, wherein initial setting times increased from 122 min (5% calcined PLA) to 158 min (30% calcined PLA), and final setting times rose from 235 to 293 min. The extension in setting times for both ash-modified cement composites is predominantly attributed to the dilution of cement clinker and the inherently slower pozzolanic activity of the ashes, which collectively impede the rate of hydration reactions. However, a consistent trend across all replacement levels reveals that cement composites containing calcined POLA exhibit longer setting times relative to those incorporating calcined PLA. This divergence is likely influenced by the respective mineralogical compositions of the ashes. Calcined POLA comprises a higher proportion of inert crystalline constituents such as quartz and hematite, which are known to delay the hydration of cementitious phases. In contrast, calcined PLA exhibits greater quantities of reactive phases, including mullite and cristobalite, which may enhance early-age pozzolanic interaction. Therefore, while both ashes function as retarders in the hydration process when compared to the control mix, calcined POLA demonstrates a more substantial delaying effect than calcined PLA.

These findings both align with and diverge from trends observed in other studies involving biomass-based ash. For instance, Khan et al. [16] reported that increasing the content of tobacco stem ash in cement paste reduced the setting times, With the initial and final times decreasing from 76 to 58 min and 182 to 160 min, respectively, at 15% replacement. In contrast, Frías et al. [18] found that BLA had minimal impact, showing only a slight delay at 20% substitution. This limited effect was likely due to its low CaO content (4.98%), which restricts early hydration. Similarly, Murali et al. [38] observed that substituting 5–40% of *Dillenia suffruticosa* ash in cement delayed the setting times compared to the control mix, With initial and final setting times ranging from 83 to 98 min and 124–155 min, respectively. For mixes containing *Acacia auriculiformis* ash, the initial and final setting times ranged from 88 to 108 min and 131–170 min, respectively. These results indicate that the effect of plant-based ash on the setting time of cement composites is highly dependent on the specific type of ash used and can vary significantly between plant sources.

### Flowability of fresh cement composite

**Fig. 10:**
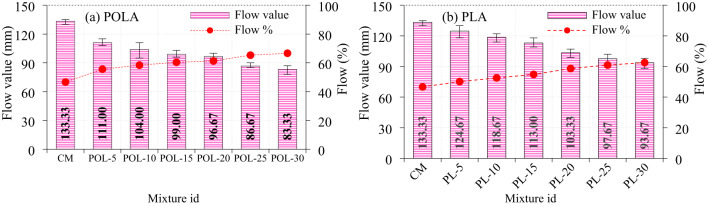
Flowability of fresh cement composite (**a**) P calcined OLA and (**b**) calcined PLA.

A comprehensive evaluation of the flow characteristics of cement composite mixtures incorporating calcined POLA and calcined PLA as partial replacements for cement (ranging from 0% to 30%) demonstrated distinct behavioral patterns governed by the physicochemical properties of the ashes and their respective incorporation levels, as illustrated in Fig. 10. The control mixture (CM) yielded a flow diameter of 133.33 mm, thereby serving as a benchmark for assessing the impact of ash substitution on workability. Upon the introduction of 5% ash, the calcined PLA-modified mixture exhibited a relatively modest reduction in flow to 124.67 mm, whereas the corresponding calcined POLA-based mixture experienced a more significant decrease to 111.00 mm. This indicates that calcined PLA exhibits better compatibility With the cementitious matrix, particularly in terms of preserving flowability at low substitution levels. At the 10% replacement level, this trend persisted, With calcined PLA achieving a flow diameter of 118.67 mm, while calcined POLA further declined to 104.00 mm. At 15% replacement, the reduction in flow became more pronounced for both systems; however, the calcined PLA mixture maintained superior flowability at 113.00 mm, as opposed to the calcined POLA mixture, which dropped to 99.00 mm, reinforcing calcined PLA’s beneficial role in mitigating flow loss. Although the difference between the two ashes diminished slightly at the 20% substitution level, calcined PLA continued to outperform calcined POLA, With flow values of 103.33 mm and 96.67 mm, respectively, underscoring its rheological stability at higher replacement rates.

A further decline in workability was observed at 25%, particularly for the calcined POLA-based system, which registered a flow of 86.67 mm, in contrast to calcined PLA’s relatively higher value of 97.67 mm. Notably, at 30% replacement, the calcined PLA mixture showed a slight increase in flow diameter to 93.67 mm, possibly due to diminished reactivity or altered fineness, whereas the calcined POLA mix exhibited continued reduction to 83.33 mm. These observations collectively confirm the superior flow-retention capability of calcined PLA across all replacement levels, likely attributed to its favorable physical texture, lower absorptivity, and enhanced interaction with the cementitious matrix. The superior flowability of calcined PLA-based cement composite compared to calcined POLA-based mixes is primarily due to the finer, more spherical particles of PLA, which enhance packing efficiency and reduce interparticle friction. In contrast, calcined POLA’s coarser, irregular particles with higher specific surface area attributed to porous carbonaceous content demand more water for lubrication, diminishing workability. Calcined PLA’s lower SSA minimizes water demand, resulting in better dispersion and improved rheological behavior. Murali et al^26^. detected that elevating the content of *Dillenia suffruticosa* and *Acacia auriculiformis* ash in cement composite mixtures led to reduced flowability, With flow values decreasing from 159.17 mm to 129.50 mm for *Dillenia suffruticosa* (0–40%) and from 148.67 mm to 110.17 mm for *Acacia auriculiformis* (5–40%), indicating a decline in workability with higher ash replacement levels. Cement composites with higher specific surface areas, composed of finer particles, offer more sites for water-cement interaction, enhancing reactivity but increasing water demand. This often requires a higher water-to-cement ratio to maintain workability, leading to improved flowability and higher flow values^26^. Conversely, ashes with lower specific surface areas contain coarser particles, limiting water interaction and reducing cement composite flow^13,26,40^.

### Effect of calcined POLA on the compressive strength of mortar cubes

**Fig. 11:**
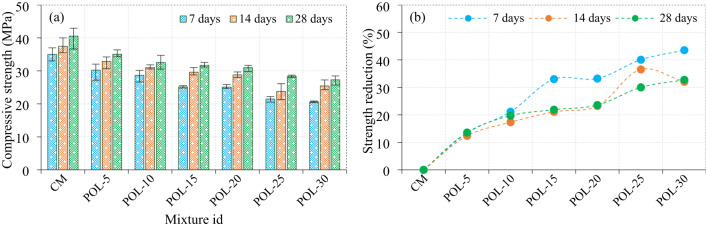
Compressive strength of calcined POLA specimens.

The compressive strength progression of specimens incorporating calcined POLA as a partial cement replacement, ranging from 5% to 30%, is illustrated in Fig. 11. The CM, containing no calcined POLA, attained compressive strengths of 35.03 MPa, 37.49 MPa, and 40.54 MPa at 7, 14, and 28 days, respectively. In contrast, the inclusion of calcined POLA led to a systematic decline in compressive strength With increasing replacement levels. At 7 days, the strength values decreased progressively from 30.22 MPa for the POL-5 mix to 20.65 MPa for the POL-30 mix, indicating reductions ranging between 13.41% and 43.54% in comparison to the control. A similar trend was detected at 14 days, With strengths declining from 32.86 MPa to 25.47 MPa (representing a 12.35% to 32.05% reduction), and at 28 days from 35.03 MPa to 27.27 MPa (With reductions between 13.58% and 32.72%). The POL-5 mixture, With 5% calcined POLA substitution, demonstrated moderate drops in compressive strength across all curing ages 13.41%, 12.35%, and 13.58% at 7, 14, and 28 days, respectively suggesting minimal adverse influence on early- and late-age strength. In contrast, the POL-10 mix exhibited more significant strength losses of 21.11%, 17.32%, and 19.68% at corresponding ages, indicating a growing impact on mechanical performance. This downward trend continued with the POL-15 mixture, which showed reductions of 32.97%, 21.07%, and 21.87%, further intensifying with POL-20 at 33.19%, 23.22%, and 23.57%. The most substantial reductions were observed in POL-25 and POL-30 specimens, With the former experiencing compressive strength losses of 40.05%, 36.51%, and 30.01%, and the latter exhibiting the highest reductions of 43.54%, 32.05%, and 32.72% at 7, 14, and 28 days, respectively. The decline in compressive strength exhibited by sample can be ascribed to the predominance of inert or minimally reactive mineral phases, notably quartz and mullite, which exhibit negligible participation in the development of C-S-H gels^41^. Furthermore, the relatively low abundance of pozzolanic constituents such as anorthite and crystalline clay phases limits secondary hydration reactions^26^. This mineralogical composition is validated by the XRD analysis of calcined PLA, as illustrated in Fig. 7a.The persistence of unreacted portlandite, coupled with elevated calcite content, suggests suboptimal hydration kinetics and insufficient binder gel formation, thereby contributing to increased porosity and reduced structural integrity of the matrix^42^.

### Effect of calcined PLA on the compressive strength of mortar cubes

**Fig. 12:**
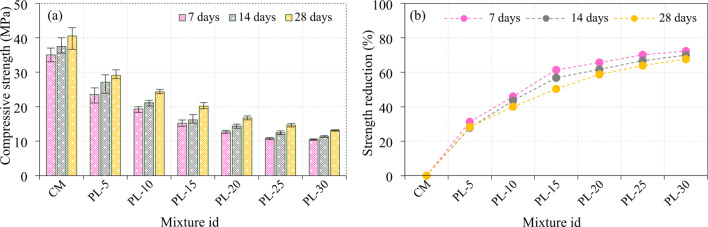
Compressive strength of calcined PLA specimens.

The evolution of compressive strength in cement composite specimens containing calcined PLA as a supplementary cementitious material, incorporated at replacement levels ranging from 5% to 30%, is detailed in Fig. 12. An inverse correlation was consistently observed between the calcined PLA dosage and compressive strength at all curing ages 7, 14, and 28 days signifying a detrimental effect of elevated calcined PLA content on the mechanical behavior of the cement composite matrix. At the 7-day mark, the compressive strength exhibited a marked decline from 23.55 MPa for the PL-5 mixture to 10.45 MPa for the PL-30 blend, corresponding to reductions of 31.27% to 72.27% relative to the control. This downward trend persisted throughout the curing period, With compressive strength values at 14 days decreasing from 27.07 MPa to 11.29 MPa (27.78%–69.87% reduction), and at 28 days from 29.10 MPa to 13.12 MPa (28.21%–67.64% reduction), reinforcing the adverse influence of higher calcined PLA dosages. Among the evaluated mixtures, the PL-5 blend exhibited the lowest degree of strength deterioration, registering compressive strength losses of 31.27%, 27.78%, and 28.21% at 7, 14, and 28 days, respectively. These relatively modest reductions suggest that a substitution rate of 5% may be considered acceptable in maintaining the structural viability of the cement composite matrix. However, the PL-10 composition demonstrated a marked escalation in strength reduction, With values decreasing by 45.89%, 43.55%, and 40.05% across the respective curing periods, suggesting that this replacement level represents a critical threshold beyond which the structural performance is substantially compromised. Substitution levels beyond this point (PL-15 and PL-20) resulted in progressively severe degradation. The PL-15 mix experienced compressive strength losses of 61.41%, 56.80%, and 50.32%, while PL-20 showed corresponding reductions of 65.65%, 61.66%, and 58.77% at the three curing intervals. The most substantial deterioration was recorded in the PL-25 and PL-30 specimens. The PL-25 mixture exhibited reductions of 70.08%, 66.73%, and 63.92%, whereas the PL-30 mix demonstrated the highest observed losses at 72.27%, 69.87%, and 67.64% for the 7, 14, and 28-day curing periods, respectively. These results indicate that calcined PLA incorporation beyond 20% significantly undermines the strength development of cement composites, likely due to reduced cementitious phase content and insufficient generation of C-S-H, which is essential for load-bearing capacity.

The presence of crystalline quartz and mullite in calcined PLA indicates a high content of inert, thermally stable phases with minimal pozzolanic reactivity^43^limiting C-S-H formation and thus dropping strength development in the cementitious matrix^26^. Despite the existence of reactive phases such as anorthite and crystalline clays, their low intensity indicates limited pozzolanic content, thereby reducing the development of strength-contributing products like C-S-H and aluminosilicate gels. This mineralogical composition is validated by the XRD analysis of calcined PLA, as illustrated in Fig. 7b.

### Comparison of compressive strength of mortar cubes incorporating calcined POLA and calcined PLA

**Fig. 13:**
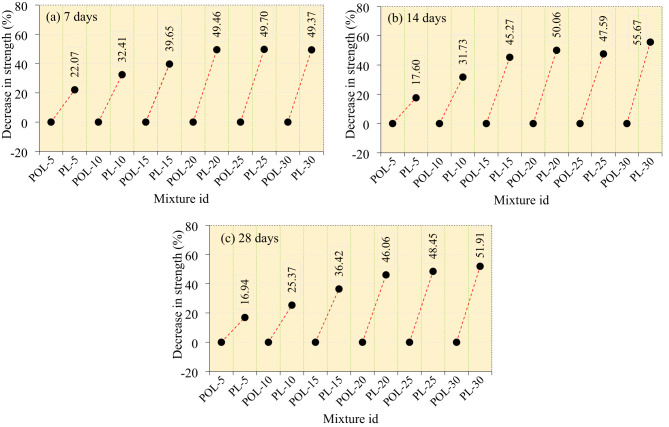
Comparison of strength between the calcined POLA and calcined PLA specimens.

The compressive strength reduction patterns of cement composite mixtures incorporating calcined POLA and calcined PLA at varying substitution levels (5% to 30%) were evaluated at 7, 14, and 28 days, as depicted in Fig. 13. At 7 days, the early-age performance clearly indicated that the incorporation of calcined POLA had a less detrimental effect on strength than calcined PLA. The POL-5 mixture showed negligible strength reduction, suggesting that a 5% replacement with calcined POLA does not compromise early compressive strength. In contrast, the PL-5 mixture exhibited a 22.07% reduction, reflecting a more pronounced negative impact even at minimal substitution. As the replacement level increased, both ash types led to progressive declines in strength, With calcined PLA showing consistently higher losses. For instance, at 30% replacement, PL-30 and POL-30 exhibited reductions of 49.37% and 49.70%, respectively, confirming the severity of strength loss at high ash contents. However, calcined PLA replacements at all levels resulted in marginally higher strength penalties compared to their calcined POLA counterparts, indicating slower pozzolanic activity and potential interference With early hydration. At 14 days, the ongoing hydration and pozzolanic reactions slightly improved strength retention for lower substitution levels. The POL-5 mixture continued to show almost no significant loss in strength, while the PL-5 mixture recorded a 17.60% reduction, again demonstrating calcined POLA’s superior compatibility at this dosage. As the substitution level increased, the strength loss became more pronounced in both series. Calcined PLA-based mixtures, particularly PL-15 (45.27%) and PL-30 (55.67%), displayed significantly higher reductions compared to POL-15 (31.73%) and POL-30 (47.59%), indicating a delay or deficiency in reactivity. Notably, the strength losses for calcined POLA mixtures beyond 20% substitution remained under 50%, whereas calcined PLA mixtures frequently exceeded this threshold, underscoring the limitations of calcined PLA in maintaining mid-age strength development. By 28 days, the influence of pozzolanic activity became more evident. POL-5 demonstrated a strength reduction of only 16.94%, affirming its long-term performance stability. Calcined PLA at the same substitution level showed an equivalent 16.94% loss, suggesting eventual strength gain through delayed pozzolanic contributions. However, for higher replacement levels, calcined POLA again proved more efficient. At 15%, POL-15 and PL-15 experienced reductions of 36.42% and 36.42%, respectively. The gap widened for the highest dosage, where POL-30 and PL-30 showed losses of 51.91% and 51.91%, respectively. These results demonstrate that although calcined PLA catches up in strength retention over time, it consistently underperforms compared to calcined POLA, particularly at early and mid-curing stages.

The evaluation of compressive strength across cementitious systems incorporating various plant leaf ashes namely *Acacia auriculiformis*, *Dillenia suffruticosa*, Bermuda grass, bamboo, calcined POLA, and calcined PLA reveals marked differences in performance over substitution levels ranging from 0% to 30% (Table 2). In the absence of ash (0% replacement), control specimens recorded compressive strengths from 33.98 MPa for *Dillenia suffruticosa* to 46.84 MPa for *Acacia auriculiformis*^26^. Among the studied ashes, *Acacia auriculiformis* consistently exhibited the highest mechanical strength, attaining a peak of 57.18 MPa at 5% incorporation. This enhancement is presumably due to its elevated pozzolanic activity and favorable particle characteristics, which likely promote better particle packing and improved microstructural development. Likewise, *Dillenia suffruticosa* demonstrated a considerable increase in compressive strength, reaching 59.67 MPa at 10% replacement, indicative of effective pozzolanic reactivity at intermediate dosages^26^. Conversely, the addition of Bermuda grass ash caused a progressive reduction in strength values, declining from 38.50 MPa at 0% to 19.81 MPa at 20%, suggesting inadequate chemical reactivity and potentially unfavorable particle morphology that could hinder hydration processes and matrix cohesion^17^. Bamboo ash displayed relatively stable compressive strength over the tested range, With only minor reductions observed from 40.54 MPa in the control to values between 36.5 MPa and 38.0 MPa at higher replacement levels suggesting moderate pozzolanic behavior capable of preserving structural integrity^36^. In contrast, in this current study, both calcined POLA and calcined PLA were associated With a noticeable decline in compressive strength as partial replacement levels increased. Calcined POLA showed a gradual decrease from 35.03 MPa at 5% to 27.27 MPa at 30%, while calcined PLA experienced a more pronounced reduction from 29.10 MPa to 13.12 MPa over the same interval. These trends imply that plant-derived ashes contribute limited pozzolanic benefits and may act as inert fillers or induce dilution effects due to the presence of non-reactive components. The more significant strength loss observed with calcined PLA suggests less favorable physicochemical properties relative to calcined POLA. This indicates that at higher partial replacement levels, both calcined POLA and calcined PLA exhibit limited pozzolanic contribution, as shown by the gradual decline in compressive strength, with calcined PLA experiencing a more pronounced reduction, suggesting weaker chemical reactivity and a higher content of inert materials that compromise matrix strength.

**Table 2:** A comparative evaluation of the mortar compressive strength of calcined POLA and calcined PLA was conducted against other types of plant leaf ashes.

% of leaf ash	^26^		^17^	^36^	In our study	
	Acacia auriculiformis	Dillenia suffruticosa	Bermuda grass	Bamboo	Calcined POLA	Calcined PLA
0	46.84		33.98	38.50	40.54	
5	57.18	58.27	32.03	-	35.03	29.10
10	54.90	59.67	26.62	37.50	32.56	24.30
15	50.97	57.86	23.83	-	31.67	20.14
20	48.10	52.51	19.81	38.00	30.98	16.71
25	47.50	51.35	-	-	28.37	14.63
30	44.57	50.59	-	36.50	27.27	13.12

### Effect of calcined POLA and calcined PLA on the water absorption of cement composite

**Fig. 14:**
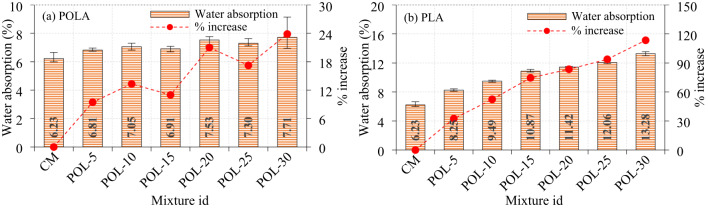
Water absorption of mortar cubes.

Cement composite specimens incorporating calcined POLA at 0–30% partial replacement levels exhibited increased water absorption with higher ash content, indicating greater porosity (Fig. 14a). The CM With 100% OPC showed the lowest absorption at 6.23%. At 5% calcined POLA (POL-5), absorption increased to 6.81% (9.45% rise), likely due to microstructural voids and delayed hydration from calcined POLA’s lower reactivity. A further increase to 10% calcined POLA (POL-10) resulted in 7.05% absorption (13.30% rise), suggesting that calcined POLA’s filler effect was inadequate to offset reduced OPC, With its fine, irregular particles possibly raising water demand and porosity. At 15% calcined POLA replacement (POL-15), water absorption slightly decreased to 6.91%, suggesting an optimal balance between pozzolanic activity and particle packing, where fine ash particles improved microstructural density. However, beyond this level, water absorption increased markedly by 7.53% for POL-20, 7.30% for POL-25, and 7.71% for POL-30 indicating 20.98%, 17.22%, and 23.84% rises from the control, respectively. These results suggest that calcined POLA contents above 15% may dilute the cement matrix, reduce available calcium hydroxide for pozzolanic reactions, and increase porosity due to excess unreacted ash. The variation in water absorption With increasing calcined POLA content is influenced by the filler effect, pozzolanic activity, and dilution effect. At replacement levels up to 15%, calcined POLA enhances particle packing and promotes secondary C-S-H development, enhancing the packing efficiency of the microstructure. However, at levels of 20% and above, cement dilution and incomplete ash reactivity lead to increased porosity. Additionally, the elevated surface area and angular morphology of calcined POLA contribute to an increased water demand in the mix, potentially affecting compaction. Thus, while moderate calcined POLA use improves matrix density, excessive replacement deteriorates pore structure and may impair long-term durability.

Cement composite specimens incorporating calcined PLA as a partial substitute for cement, across partial replacement levels ranging from 0% to 30%, exhibited a consistent increase in water absorption with rising ash content, like the trend observed with calcined POLA (Fig. 14b). At a 5% calcined PLA replacement (PL-5), the water absorption increased to 8.25%, representing a 32.46% increase compared to the control mix. A subsequent rise in PLA content to 10% (PL-10) resulted in a water absorption of 9.49%, reflecting a 52.40% rise, indicating that the filler effect of calcined PLA was insufficient to compensate for the reduction in OPC content. The fine, irregular morphology of calcined PLA particles likely contributed to an increase in water demand and porosity. At 15% calcined PLA replacement (PL-15), water absorption rose further to 10.87%. Beyond this threshold, the water absorption increased noticeably, With values of 11.42% for PL-20, 12.06% for PL-25, and 13.28% for PL-30, indicating increases of 83.50%, 93.72%, and 113.9%, respectively, relative to the control mix. An increase in the proportion of calcined PLA results in a corresponding reduction in the quantity of OPC available to participate in the hydration process. The hydration products of OPC, particularly C-S-H are critical in enhancing strength and reducing the permeability of the cement composite. Consequently, a decrease in OPC content contributes to diminished C-S-H phase development, which led to less dense and more porous microstructure, thereby increasing the water absorption capacity of the cement composite. Compared to the calcined POLA, the calcined PLA exhibited higher water absorption. This occurrence results from the calcined PLA having a finer, more irregular particle structure than calcined POLA, which increases its surface area and water demand during mixing. This higher water requirement facilitates the hydration of ash particles, resulting in a more porous microstructure and greater water absorption in the hardened cement composite.

### Failure pattern of the cubes under compression

**Fig. 15:**
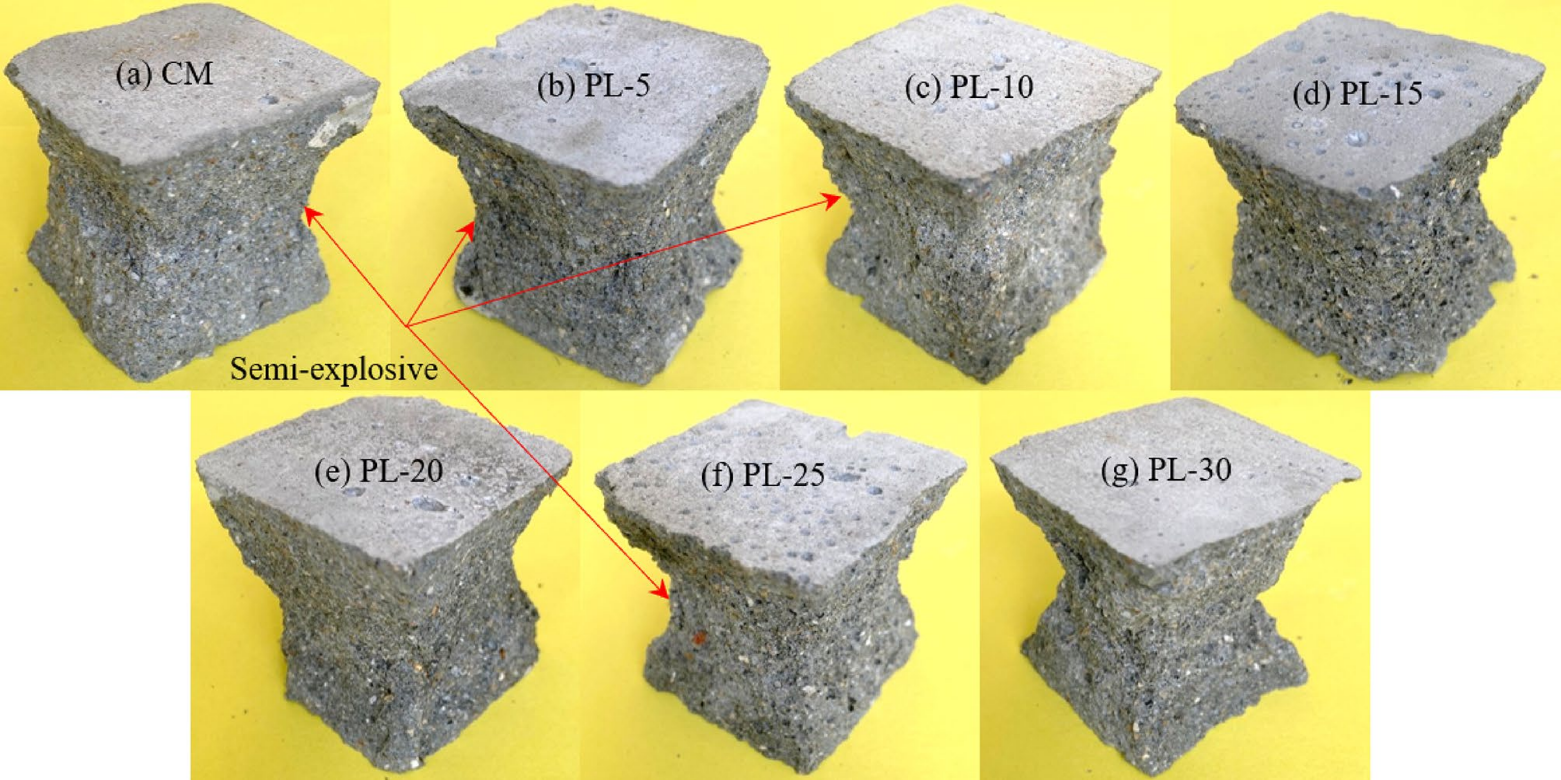
Failure pattern of the specimens with varying calcined PLA contents.

The control specimen demonstrated a well-defined and uniform shear failure, characterized by sharp and continuous crack propagation (Fig. 15a), indicative of a compact and homogeneous microstructural arrangement^26^. The absence of calcined PLA facilitated unobstructed cement hydration and the development of a cohesive matrix, thereby producing a predictable failure mode under compressive loading. The inclusion of 5% calcined PLA exhibited negligible influence on the fracture behavior when compared to the control mix (Fig. 15b). The specimen retained its mechanical integrity, suggesting that the low-level replacement did not significantly impair the reactivity of the binder system. Slight surface anomalies may be attributed to minor disturbances in granular arrangement or internal void distribution. At a 10% partial substitution level, deviations in failure morphology began to emerge, with the appearance of non-uniform fracture planes and marginal bulging along the cube edges (Fig. 15c). These signs may reflect partial pozzolanic activity and the presence of unreacted calcined PLA particles, contributing to marginal disruptions in matrix compaction. The 15% calcined PLA partial replacement specimen (Fig. 15d) exhibited noticeable surface blistering and localized crushing at the corners, suggesting a decline in microstructural integrity. At this concentration, the reduction in active cementitious constituents appears to outweigh the limited reactivity of calcined PLA, resulting in suppressed strength development.

A further specimen incorporating 15% calcined PLA (Fig. 15e) revealed a distinctly irregular fracture profile accompanied by prominent edge spalling. This indicates a transition to a more brittle failure mechanism, likely arising from diminished cohesion within the matrix. The limited availability of calcium hydroxide for secondary hydration reactions adversely affected the formation and stability of the interfacial transition zone (ITZ)^26^leading to mechanical degradation. With a 25% calcined PLA partial substitution, failure became markedly brittle, exhibiting visible surface voids and rugged fracture surfaces (Fig. 15f). This deterioration in structural performance is likely due to the excessive calcined PLA acting predominantly as a non-reactive filler, thereby interrupting matrix continuity and compromising stress distribution efficiency. At the highest partial replacement level of 30%, the composite underwent significant structural disintegration, marked by a highly irregular fracture mode and notable delamination of the top section from the body of the cube (Fig. 15g). These findings suggest pronounced matrix porosity and weak interparticle bonding^26^. The excessive incorporation of calcined PLA at this stage severely hindered the matrix’s ability to sustain mechanical loads, underscoring the detrimental effect of high-volume calcined PLA replacement on compressive strength and overall material stability.

### Mechanism of action

**Fig. 16:**
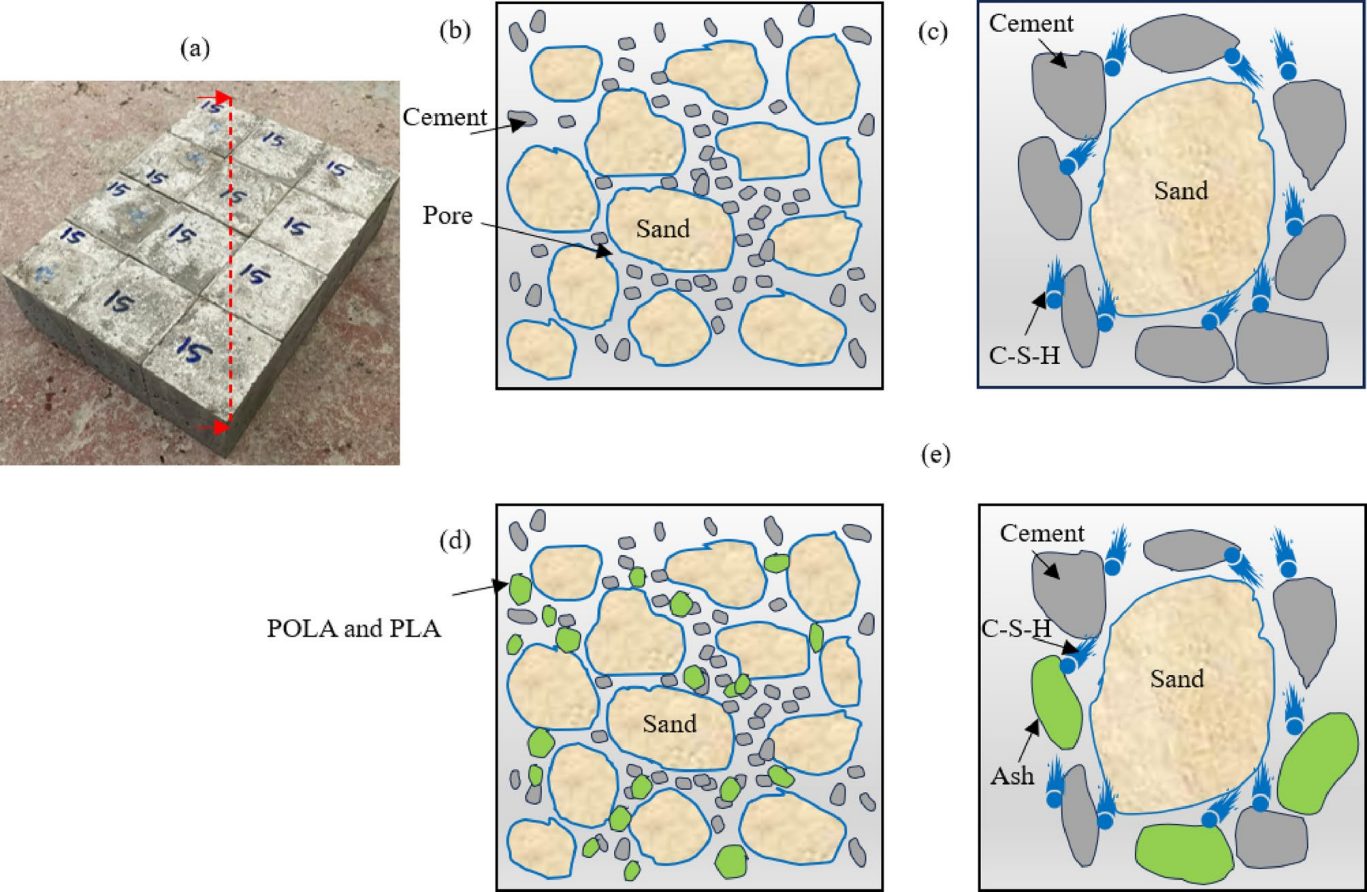
Mechanistic representations of the effects induced by ash substitution in cementitious systems is illustrated through: (**a**) a standard mortar cube, (**b**) conventional cement composite composition, (**c**) the fundamental hydration reactions of cement, (**d**) modified cement composite incorporating plant leaf ash, and (**e**) the altered hydration mechanisms resulting from the interaction between leaf ash and cementitious phases. (Concept adopted and reproduced from^26,44^.

Figure 16 illustrates the hydration mechanism in cement partially replaced with calcined POLA and calcined PLA. As seen in Figs. 16 (b) and (c) the unmodified cement matrix exhibits voids caused by water separation at particle interfaces^26,44^. During hydration, cement undergoes hydration, resulting in the formation of C-S-H, an amorphous gel that fills interstitial voids, enhancing matrix compactness and strength. The dissolution of ions from cement surfaces sustains hydration and promotes further matrix densification. Consequently, cement particles exhibit improved uniformity in distribution, reducing agglomeration and improving reactivity. Figures 16 (d) and (e) highlight the larger particle sizes of POLA and PLA ashes compared to cement. Their coarser texture and higher surface area improve particle packing but also increase water absorption, which can delay initial hydration. Thermal activation imparts pozzolanic reactivity, enabling secondary C-S-H formation at later curing stages^26,45^. However, the higher water demand for workability raises the water-to-cement ratio, increasing porosity and microcrack formation, which may lower compressive strength^44^. Despite this, leaf ash incorporation helps moderate the heat of hydration, which supports its application in large-scale concrete elements exposed to the risk of thermal cracking^26^.

## Microstructure

### SEM analysis of cement composite sample containing calcined POLA and calcined PLA

**Fig. 17:**
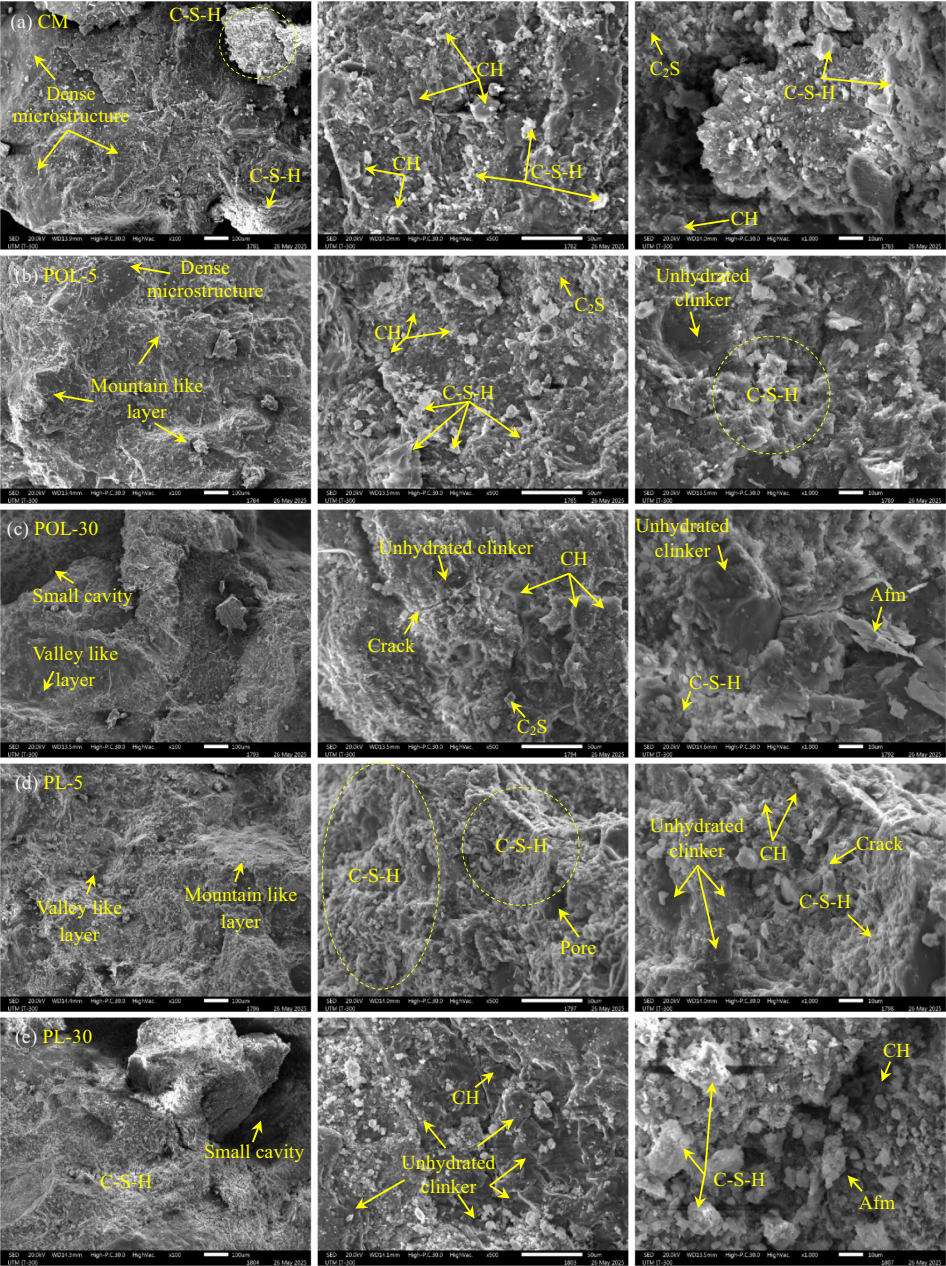
SEM analysis of cement composite sample.

The microstructural characteristics of cement composites incorporating POL ash and PL at varying replacement levels were systematically examined using SEM (Fig. 17). The control specimen, illustrated in Fig. 17(a), revealed a well-compacted and homogeneous matrix predominantly composed of abundant C-S-H phases alongside distinctly layered calcium hydroxide (CH) crystals. This morphology is indicative of a fully progressed hydration process and an optimized cementitious framework^26,46^. Upon substitution of 5% of cement with POLA, as observed in Fig. 17(b), the matrix retained its densified structure, exhibiting a coarse-textured, mountainous surface with widespread and uniform C-S-H gel deposition. Similar findings reported by the study^26^cement composite specimens With 10% *Dillenia suffruticosa* and 40% *Acacia auriculiformis* leaf ash exhibited a layered granular structure, forming a surface profile akin to undulating terrain. A discernible reduction in CH crystal content, in comparison to the control matrix, points toward the initiation of pozzolanic reactions, where reactive silica and alumina existing in calcined POLA involve in secondary hydration processes, consuming CH ^44^. Conversely, at an increased substitution rate of 30% calcined POLA (Fig. 17c), the microstructure transitioned into a more porous and discontinuous form. SEM images revealed microcracking, unreacted dicalcium silicate (C_2_S) remnants, and enlarged voids, collectively implying suboptimal hydration kinetics and limited pozzolanic interaction^47^. Such a configuration reflects a compromise in microstructural integrity and inadequate C-S-H formation, attributable to the over-replacement of cementitious material With ash of lower reactivity. A parallel trend was evident in specimens containing calcined PLA. At a 5% replacement level (Fig. 17d), the cement composite presented a moderately refined microstructure, distinguished by undulating topography With both ridge-like and valley-like features. Although C-S-H development was confirmed, the occurrence of surface irregularities and isolated porosity suggested a marginally slower hydration progression relative to that of the POL-based counterpart. In the case of the 30% PL-substituted specimen (Fig. 17e), SEM analysis identified sparse hydration products, numerous small cavities, unhydrated cement clinker, and dispersed CH crystals, all indicative of incomplete cement hydration and a weak pozzolanic response. The C-S-H phase, while present, appeared disjointed and less consolidated compared to specimens with lower PL content. Tavares et al^48^. detected CH and C-S-H phases in cement paste samples comprising BALA. In contrast, Bhutto et al^19^. documented that substituting 20% of cement with BALA significantly enhances the microstructural integrity of the interfacial transition zone, yielding a uniformly compacted matrix with negligible manifestation of voids or microcracks.

Overall, both calcined POLA and calcined PLA contributed to enhanced microstructural development when used at limited dosages (5%), primarily by stimulating secondary C-S-H generation via pozzolanic mechanisms. However, elevated replacement levels (30%) were detrimental, as the dilution of cementitious phases curtailed hydration efficacy, increased internal voids, and disrupted interfacial coherence. Noteworthy is the comparatively higher pozzolanic reactivity of POL ash at lower replacement ratios, evidenced by a more interconnected C-S-H gel network and fewer unhydrated inclusions than those observed in the PL-5 cement composite.

### XRD analysis of cement composite sample containing calcined POLA and calcined PLA

**Fig. 18:**
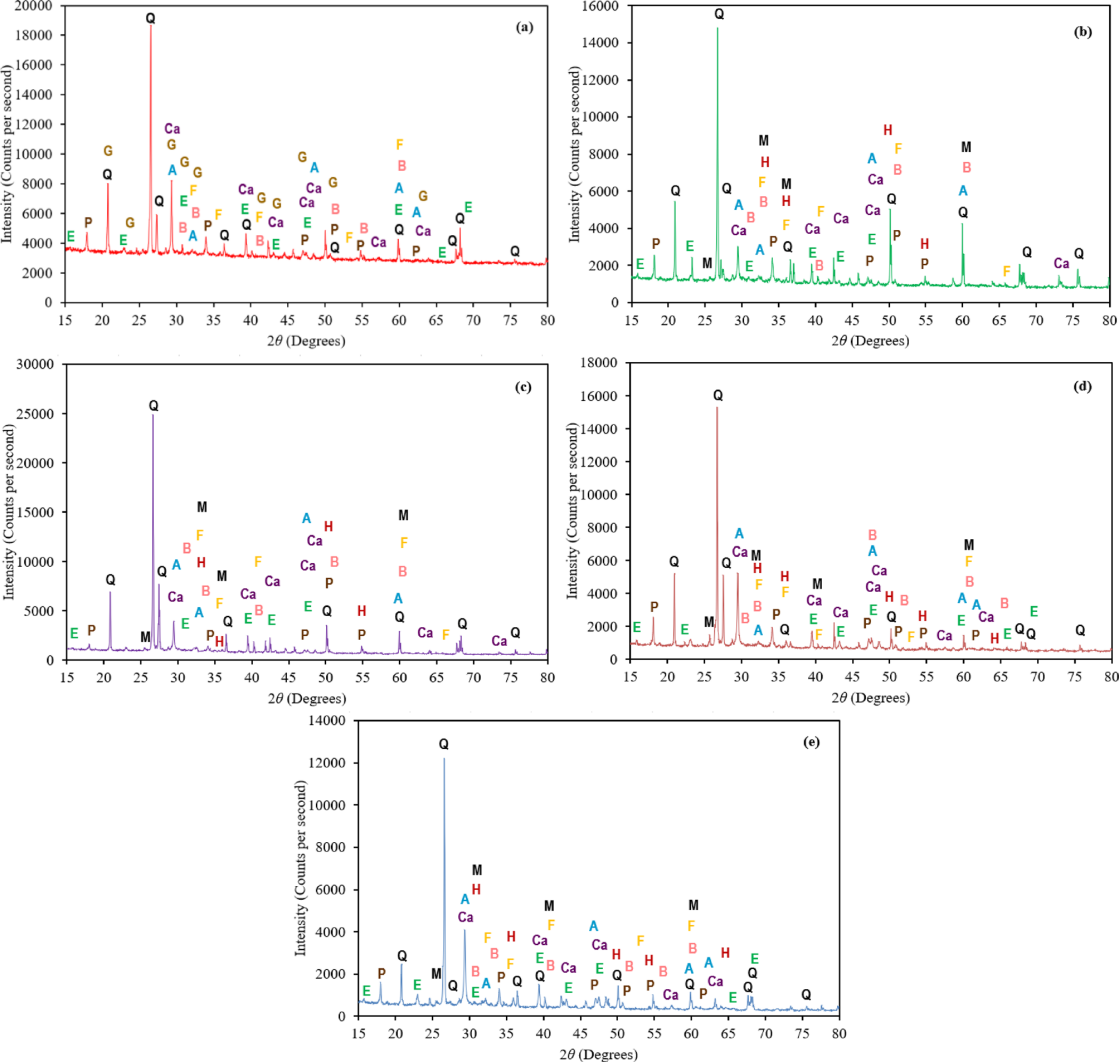
XRD test results for (**a**) control (**b**) 5% calcined POLA (**c**) 30% calcined POLA (**d**) 5% calcined PLA (**e**) 30% calcined PLA of cementitious composites. Note: Q = Quartz (SiO_2_), E = Ettringite [Ca_6_Al_2_(SO_4_)_3_(OH)_12_·27H_2_O], Ca = Calcite (CaCO_3_), A = Alite (Ca_3_SiO_5_), B = Belite (2CaO·SiO_2_), Ferrite (MO·Fe_2_O_3_), Hematite (Fe_2_O_3_), M = Mullite (Al_6_Si_2_O_13_), P = Portlandite [Ca(OH)_2_].

The XRD analysis of the control cement composite reveals a typical mineralogical composition characterized by both hydration products and unhydrated clinker phases. Prominent quartz peaks at 20.8°, 26.6°, and 27.3° identify it as an inert filler. Portlandite shows strong peaks at 18.0°, 34.1°, 47.1°, and 50.8°, indicating active hydration (Fig. 18a). Ettringite is marked by peaks at 15.8°, 22.9°, and 31.1°, reflecting early-age sulfate reactions. Calcite at 29.4° and 39.4° suggests carbonation, while persistent alite and belite, along With ferrite at 33.0° and 35.5°, confirm incomplete hydration. Incorporating 5% calcined POLA introduces additional phases and alters the intensity of existing ones (Fig. 18b). The quartz peaks remain visible at 26.6° and 20.8°, suggesting that calcined POLA contributes unreacted silica, acting primarily as a micro-filler. Portlandite peaks (18.0°, 34.1°, 54.3°) are still evident, though slightly reduced compared to the control, indicating that limited pozzolanic reactions have commenced. Ettringite remains present at 15.8°, 22.9°, and 31.1°, and calcite intensifies slightly at 29.4°, 39.4°, and 43.1°, which may be attributed to either increased carbonation or the presence of calcium carbonate in the ash. Residual clinker phases such as belite and alite persist, indicating partial hydration, while the detection of ferrite and hematite suggests iron-rich contributions from both cement and calcined POLA. Mullite peaks at 26.0°, 33.2°, and 60.8° confirm the ash’s calcination, serving as an inert, thermally stable filler. When the calcined POLA content is increased to 30%, significant shifts in mineralogical profiles occur (Fig. 18c). Quartz peaks become more intense (20.8°, 26.6°, 36.5°), indicating a substantial presence of unreacted silica, which underscores the filler role of the ash at higher dosages. The portlandite peaks at 18.0°, 34.1°, and 54.3° are notably diminished, reflecting the consumption of calcium hydroxide through pozzolanic reactions that lead to additional C-S-H formation. Ettringite remains present, confirming sustained early sulfate reactions. Intensified calcite peaks suggest elevated carbonation or the presence of calcium carbonate from calcined POLA. Persistent alite and belite peaks again point to incomplete hydration, likely due to cement dilution. Enhanced ferrite and hematite signals indicate increased iron content from the ash, while the continued presence of mullite confirms the ash’s role as a stable, non-reactive phase that contributes to microstructural refinement.

A similar trend is observed in cement composites incorporating calcined PLA. At 5% replacement, quartz peaks at 26.6° and 20.8° indicate unreacted silica from the ash, while portlandite (18.0°, 34.1°, 47.1°, 54.3°) shows moderate intensity, suggesting some early pozzolanic reactivity (Fig. 18 d). Ettringite (15.8°, 22.9°, 31.1°) and calcite (29.4°, 39.4°, 43.1°) are observed at expected positions, mirroring patterns seen in calcined POLA mixes. The presence of alite and belite highlights partial hydration, and ferrite and hematite again confirm iron contributions from calcined PLA. Mullite at 26.0°, 33.2°, and 60.8° denotes effective ash calcination. At 30% calcined PLA, the mineralogical profile shifts significantly (Fig. 18e). Strong quartz peaks (26.6°, 27.3°, 36.5°, 50.1°) dominate the diffractogram, reflecting substantial unreacted silica content. The marked reduction in portlandite peaks (18.0°, 34.1°, 47.1°, 54.3°) indicates active pozzolanic consumption of Ca(OH)₂. Persistent ettringite (15.8°, 22.9°, 31.1°) confirms that sulfate reactions continue despite reduced cement content. Intensified calcite (29.4°, 39.4°, 43.1°) peaks suggest carbonation or the direct presence of calcium carbonate in the ash. Alite and belite phases remain visible, consistent with dilution-induced hydration retardation. Iron-rich phases, including ferrite and hematite (33.0°−35.6°), are pronounced, and the detection of mullite (26.0°, 33.2°, 60.8°) affirms the thermal transformation of calcined PLA into a stable, non-reactive filler. Overall, both calcined POLA and calcined PLA cement composites exhibit mineralogical signatures indicative of pozzolanic activity and filler effects, With the extent of these effects strongly governed by the replacement level. Other studies reported that the XRD analysis of other biomasses. For instance, Khan et al. reported that in cementitious blends incorporating 7.5% tobacco stem ash, the intensity of portlandite peaks was notably elevated, indicating a higher degree of portlandite formation likely associated with accelerated hydration processes during the early curing phase^16^. Silva et al^36^. reported that increasing banana leaf ash content led to a reduction in calcium hydroxide (CH) peaks, indicating enhanced pozzolanic activity. XRD analysis showed calcite, C-S-H, and quartz phases, With intensified calcite peaks at 29.5°, suggesting improved reaction efficiency. In *Dillenia suffruticosa*-blended samples, portlandite peaks became more pronounced at 40% substitution, reflecting ongoing hydration but incomplete C–S–H formation^26^. In contrast, *Acacia auriculiformis* samples at 5% and 40% showed lower portlandite intensity, likely due to higher silica and alumina content promoting more effective pozzolanic reactions and reduced free lime.

### TGA analysis of cement composite sample containing calcined POLA and calcined PLA

**Fig. 19:**
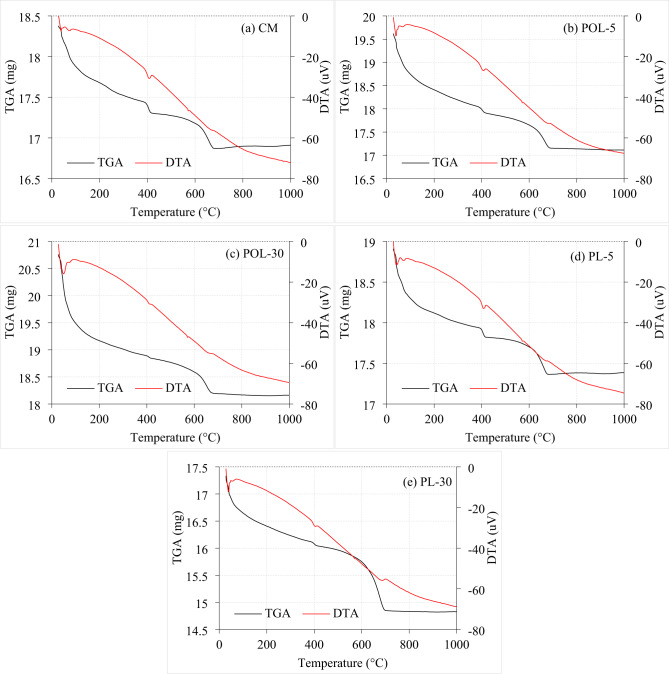
TGA analysis of cement composite sample with calcined POLA and calcined PLA.

The thermogravimetric analysis (TGA) and differential thermal analysis (DTA) performed on CM, calcined POLA and calcined PLA samples provide a comprehensive understanding of their thermal stability and pozzolanic performance. The CM sample exhibited distinct and well-defined thermal decomposition stages. The observed mass reduction Within the 100 °C to 200 °C temperature range is attributed to the evaporation of free water and physically bound moisture^48,49^followed by a more substantial weight drop between 400 °C and 500 °C due to the dehydroxylation of CH (Fig. 19a). A further decrease in mass falling between 600 °C and 750 °C is attributed to the decarbonation of CaCO₃^50^. These thermal events were mirrored by corresponding endothermic peaks in the DTA curve, confirming the presence and degradation of CH and CaCO₃ phases within the cementitious matrix. The POL-5 samples led to moderate alterations in the thermal decomposition profile (Fig. 19b). The TGA curve exhibited a slightly increased initial mass and a modest reduction in mass loss associated with both CH and CaCO₃ decomposition. These variations suggest that the pozzolanic reaction at this substitution level was limited, with only partial consumption of CH^51^. This observation is further corroborated by the DTA curve, which revealed diminished endothermic peaks compared to CM, indicating reduced quantities of CH and carbonate phases available for decomposition. However, a substantial enhancement in pozzolanic reactivity was evident in the POL-30 sample. The CH decomposition onset shifted to a lower temperature range (400–470 °C), and the mass loss corresponding to carbonate breakdown was significantly reduced (Fig. 19c). Additionally, the residual mass at 1000 °C was higher than that of both CM and POL-5, indicating the formation of increased quantities of thermally stable hydration products^35^. The DTA profile for POL-30 displayed broader and less intense endothermic peaks, signifying a more advanced pozzolanic reaction and a denser, more stable matrix. These findings suggest that higher calcined POLA replacement levels can significantly enhance the thermal resistance and long-term durability of cementitious systems.

In comparison, the thermal performance of calcined PLA samples showed similar trends, albeit with some variations in the extent of reactivity. The PL-5 mix demonstrated a thermal decomposition pattern closely aligned with that of the CM sample (Fig. 19d). Minor reductions in CH-related mass loss and slightly suppressed DTA endothermic peak suggest limited pozzolanic activity at low PLA content. However, the PL-30 mix exhibited a marked reduction in mass loss within both CH and CaCO₃ decomposition intervals, accompanied by a substantial increase in residual mass (approximately 14.8 mg at 1000 °C) (Fig. 19e). This finding indicates enhanced development of C-S-H and a denser microstructural network^26^. The DTA curve supported this inference through significantly reduced endothermic peak intensities, highlighting improved thermal stability. Critically comparing calcined POLA and calcined PLA, both ashes demonstrated increased pozzolanic activity at 30% replacement, as evidenced by lower CH and CaCO₃ decomposition and higher thermal residue. However, calcined POLA appears to initiate pozzolanic reactions more effectively at lower replacement levels (5%) compared to calcined PLA, which showed only marginal activity at the same dosage. The broader and less intense DTA peaks in POL-30 also suggest a more homogeneous and extensive pozzolanic reaction than that of PL-30. These observations collectively indicate that while both agricultural ashes can enhance the thermal stability and microstructural integrity of cement composites, calcined POLA exhibits superior performance, particularly in terms of early-stage pozzolanic activation.

### FTIR analysis of the cement composite sample containing calcined POLA and calcined PLA

**Fig. 20:**
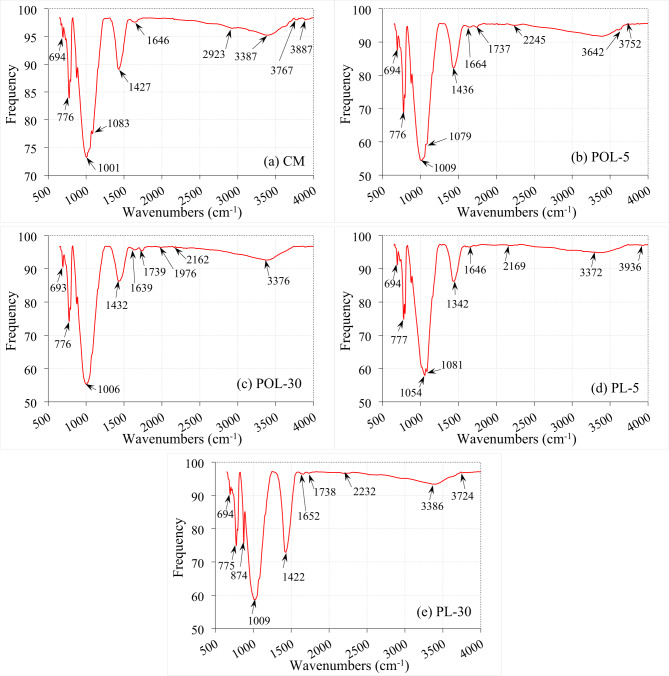
SEM analysis of cement composite sample with calcined POLA and calcined PLA.

The FTIR spectral analysis of the CM and the formulations incorporating calcined POLA and calcined PLA samples indicates discernible changes in the molecular structure and vibrational characteristics associated With ash integration. Absorption bands detected Within the spectral range of 694–777 cm⁻¹ are ascribed to Si-O bending vibrations, reflective of the structural features of C-S-H phases (Fig. 20a)^52^. Moreover, all spectra exhibit prominent peaks in the 1000–1085 cm⁻¹ region, corresponding to asymmetric stretching vibrations of the Si-O-Si bond^26,53^. A notable shift in the Si-O-Si peak from 1001 cm⁻¹ in the control sample to 1009 cm⁻¹ in the POL-5 and PL-30 specimens signifies alterations in the silicate network, likely stemming from pozzolanic activity induced by the reactive silica content of the incorporated ashes^54^. The FTIR analysis of the POL-5 reveals a series of well-defined absorption bands located at 1009, 1079, 1436, 1664, 1737, 2245, 3642, and 3752 cm⁻¹, signifying the presence of additional functional groups resulting from ash integration (Fig. 20b). The spectral bands at 1737 and 2245 cm⁻¹ are indicative of vibrational modes associated with carbonyl (C = O) and carboxyl functionalities, which are likely attributed to residual organic matter retained Within the ash matrix. Moreover, the pronounced O-H stretching vibrations observed near 3642 and 3752 cm⁻¹ suggest improved water retention capacity and an enhancement in the formation of hydration products^26^. Conversely, the POL-30 sample exhibits peaks at 1976 and 2162 cm⁻¹, in addition to a broadened O-H stretching band centered at 3376 cm⁻¹ (Fig. 20c)^55^.

PL-5 sample reveals distinct absorption bands at 1054, 1081, 1342, 1646, 2169, 3372, and 3936 cm⁻¹, indicating significant modifications in the vibrational characteristics of functional groups due to the presence of ash-derived components (Fig. 20d). The sharp band at 3936 cm⁻¹ is attributed to pronounced O-H stretching vibrations, which may signify enhanced hydration activity, potentially facilitated by the pozzolanic reactivity of calcined PLA. The emergence of a peak at 2169 cm⁻¹ is suggestive of nitrile or isocyanate functional groups, likely introduced through residual organic constituents in the ash material. Compared to calcined POLA-modified cement composites, the PL-5 specimen exhibits relatively attenuated signals in the carbonate-associated region, which may imply a lower propensity for carbonation processes. In the PL-30 formulation, incorporating a higher proportion of calcined PLA, the FTIR spectrum displays additional prominent bands at 874, 1009, 1422, 1652, 1738, 2232, 3386, and 3724 cm⁻¹ (Fig. 20e). The absorption at 874 cm⁻¹ is generally associated with increased silica reactivity, while the intensified and broadened O-H stretching bands suggest the occurrence of a delayed yet sustained hydration reaction^56^potentially influenced by the higher ash content. The incorporation of calcined POLA and calcined PLA into cementitious systems alters the chemical structure of hydration products, as indicated by FTIR spectral shifts and new absorption bands. At 5% replacement, both ashes enhance pozzolanic activity and promote the formation of silicate and hydroxyl phases. In contrast, 30% replacement leads to the retention of organic residues and reduced hydration-related signals, suggesting diminished reaction efficiency. Calcined POLA introduces carboxylic functionalities, while calcined PLA more effectively enhances silicate and hydroxyl features at lower levels. Overall, moderate ash content improves hydration and matrix densification, whereas excessive substitution impairs chemical integration^26^.

## Conclusions

This study examined the mechanical and mineralogical performance of cement composites incorporating calcined POLA and calcined PLA as SCM. The analysis of the experimental findings led to the formulation of the following principal conclusions.

1. SEM analysis revealed that calcined POLA has a coarse, porous, and layered structure, suggesting incomplete combustion and higher surface area, which may boost pozzolanic activity but limit filler uniformity. In contrast, calcined PLA displayed a finer, more uniform, microporous morphology, indicating more complete combustion and higher reactivity, making it more suitable as a supplementary cementitious material.
2. Calcined PLA-modified cement composites consistently demonstrated higher flowability than calcined POLA-based mixes across all replacement levels, With flow diameters decreasing from 124.67 mm to 93.67 mm, compared to 111.00 mm to 83.33 mm for calcined POLA. The greatest difference occurred at 25% replacement, where calcined PLA exceeded calcined POLA by 11 mm, indicating better workability retention.
3. Increasing calcined POLA replacement led to a progressive decline in compressive strength, With the highest reductions observed at 30% substitution: 43.54% at 7 days, 32.05% at 14 days, and 32.72% at 28 days compared to the control. At 5% calcined POLA replacement, strength losses were minimal 13.41% (7 days), 12.35% (14 days), and 13.58% (28 days) indicating that low substitution levels maintain acceptable strength development over time.
4. Compressive strength declined significantly With increasing calcined PLA content, reaching maximum losses at 30% replacement: 72.27% (7 days), 69.87% (14 days), and 67.64% (28 days). At 5% replacement, reductions were more moderate 31.27%, 27.78%, and 28.21%, respectively indicating potential structural viability at low substitution levels.
5. Calcined POLA-modified mixtures showed a moderate rise in water absorption, increasing from 6.23% to 7.71% at 30%, With the lowest increase at 5% (6.81%) and a slight improvement at 15% (6.91%), suggesting optimal microstructural balance. In contrast, calcined PLA mixtures exhibited significantly higher absorption, reaching 13.28% at 30%, and rising sharply to 8.25% even at 5%, consistently surpassing calcined POLA at all levels.
6. Moderate replacement (5%) of calcined POLA and calcined PLA enhances microstructure by promoting dense, homogeneous matrices with abundant C-S-H and reduced CH, indicating active pozzolanic reactions. In contrast, high replacement (30%) impairs microstructural integrity, leading to increased porosity, microcracks, and unreacted phases due to incomplete hydration and limited reactivity.
7. Calcined POLA and calcined PLA exhibit pozzolanic activity and micro-filler effects, reflected by reduced portlandite and increased quartz and mullite signals. At 5% replacement, pozzolanic reactions begin Without disrupting hydration. However, 30% replacement intensifies pozzolanic activity but reduces hydration efficiency, with diminished portlandite, unhydrated clinker phases, and signs of filler dominance and carbonation.
8. At 30% replacement, calcined POLA and calcined PLA enhance thermal stability and pozzolanic activity, shown by lower mass losses, weaker DTA peaks, and higher residual mass at 1000 °C. Calcined POLA demonstrates superior early-stage reactivity, With greater CH and carbonate reduction at 5% and broader DTA peaks at 30%, indicating more uniform pozzolanic reactions.
9. At 5% replacement, calcined POLA and calcined PLA improve pozzolanic activity and hydration, indicated by FTIR shifts in Si-O-Si bands, stronger O-H vibrations, and new functional groups. At 30%, excessive ash reduces hydration efficiency, with broader peaks and residual organics signaling incomplete reaction and poor integration.

### Scope for the future works

- Future studies should optimize calcination and post-treatment methods: such as finer grinding, acid washing, or chemical activation to reduce carbon residues and improve calcined POLA’s microstructural uniformity. Enhancing combustion conditions may also minimize incomplete combustion effects and boost reactivity.
- Co-utilizing calcined POLA and calcined PLA with other pozzolans (e.g., fly ash, silica fume, metakaolin and slag) may offset individual drawbacks, enhance filler efficiency, and balance early and long-term performance. Synergistic blends could yield SCMs with improved strength, durability, and workability.
- Long-term durability of calcined POLA- and calcined PLA-modified cement composites should be evaluated under sulfate attack, chloride ingress, carbonation, and freeze-thaw cycles to assess their suitability for structural use and understand pore structure evolution, degradation, and sustained pozzolanic activity.

## Data Availability

The datasets used in the current study are available from the corresponding author upon reasonable request.
